# Comparative genome analysis of 19 *Ureaplasma urealyticum* and *Ureaplasma parvum* strains

**DOI:** 10.1186/1471-2180-12-88

**Published:** 2012-05-30

**Authors:** Vanya Paralanov, Jin Lu, Lynn B Duffy, Donna M Crabb, Susmita Shrivastava, Barbara A Methé, Jason Inman, Shibu Yooseph, Li Xiao, Gail H Cassell, Ken B Waites, John I Glass

**Affiliations:** 1 J. Craig Venter Institute, 9704 Medical Center Drive, Rockville, MD 20850, USA; 2 University of Alabama at Birmingham, 1530 3rd Ave. S., Birmingham, AL, 35294, USA

## Abstract

**Background:**

*Ureaplasma urealyticum* (UUR) and *Ureaplasma parvum* (UPA) are sexually transmitted bacteria among humans implicated in a variety of disease states including but not limited to: nongonococcal urethritis, infertility, adverse pregnancy outcomes, chorioamnionitis, and bronchopulmonary dysplasia in neonates. There are 10 distinct serotypes of UUR and 4 of UPA. Efforts to determine whether difference in pathogenic potential exists at the ureaplasma serovar level have been hampered by limitations of antibody-based typing methods, multiple cross-reactions and poor discriminating capacity in clinical samples containing two or more serovars.

**Results:**

We determined the genome sequences of the American Type Culture Collection (ATCC) type strains of all UUR and UPA serovars as well as four clinical isolates of UUR for which we were not able to determine serovar designation. UPA serovars had 0.75−0.78 Mbp genomes and UUR serovars were 0.84−0.95 Mbp. The original classification of ureaplasma isolates into distinct serovars was largely based on differences in the major ureaplasma surface antigen called the multiple banded antigen (MBA) and reactions of human and animal sera to the organisms. Whole genome analysis of the 14 serovars and the 4 clinical isolates showed the *mba* gene was part of a large superfamily, which is a phase variable gene system, and that some serovars have identical sets of *mba* genes. Most of the differences among serovars are hypothetical genes, and in general the two species and 14 serovars are extremely similar at the genome level.

**Conclusions:**

Comparative genome analysis suggests UUR is more capable of acquiring genes horizontally, which may contribute to its greater virulence for some conditions. The overwhelming evidence of extensive horizontal gene transfer among these organisms from our previous studies combined with our comparative analysis indicates that ureaplasmas exist as quasi-species rather than as stable serovars in their native environment. Therefore, differential pathogenicity and clinical outcome of a ureaplasmal infection is most likely not on the serovar level, but rather may be due to the presence or absence of potential pathogenicity factors in an individual ureaplasma clinical isolate and/or patient to patient differences in terms of autoimmunity and microbiome.

## Background

Ureaplasmas belong to the class *Mollicutes*. Like other members of this class, which are obligate parasites of eukaryotes, ureaplasmas lack a cell wall, use a non-standard genetic code, have extremely small genome size, and require cholesterol. There are five species in the class *Mollicutes* that are human pathogens. The best known is *Mycoplasma pneumoniae*, which is a respiratory pathogen that is an agent of “walking pneumonia.” The other four, *Mycoplasma genitalium*, *Ureaplasma parvum* (UPA), *Ureaplasma urealyticum* (UUR), and *Mycoplasma hominis* are all urogenital pathogens. Ureaplasmas are among the smallest self-replicating organisms capable of a cell-free existence. They were described first in 1954 [1] and the genus *Ureaplasma* was established in 1974 [2], comprising those members of the family *Mycoplasmataceae* that hydrolyze urea and use it as a metabolic substrate for generation of ATP. This genus currently has seven recognized species that have been isolated from humans and various animals (dogs, cats, chickens, and cattle). To date, at least 14 serovars have been identified: UUR comprises 10 serovars-UUR2, UUR4, UUR5, UUR7-13 and UPA includes 4 serovars-UPA1, UPA3, UPA6, UPA14 [3-9]. Although ureaplasmas are common commensals in healthy individuals, they are also implicated in a variety of clinical outcomes including but not limited to non-gonococcal urethritis, pelvic inflammatory disease, infertility, adverse pregnancy outcomes, chorioamnionitis and bronchopulmonary dysplasia in neonates [10]. As many as 40%–80% of healthy adult women may harbor ureaplasmas in their cervix or vagina. The infection is readily transmitted venereally as well as vertically; with a transmission rate to infants born to colonized mothers as high as 90% [10]. Their occurrence is somewhat less in the lower urogenital tract of healthy men (approximately 20%–29%) [11,12]. UPA is more common than UUR as a colonizer of the male and female urogenital tracts and in the neonatal respiratory tract [10]. Ureaplasmas reside primarily on the mucosal surfaces of the urogenital tracts of adults or the respiratory tracts in infants. They are capable of attaching to a variety of cell types such as urethral epithelial cells, spermatozoa, and erythrocytes [12]. The adhesins of ureaplasmas have not been characterized completely, but current evidence suggests the receptors are sialyl residues and/or sulphated compounds [13]. A major family of surface proteins, the multiple banded antigens (MBA), is immunogenic during ureaplasmal infections. MBAs have been used as a basis for the development of reagents for diagnostic purposes and for serotyping [11,12,14,15]. Although there is no evidence ureaplasmas produce toxins, they do possess several potential virulence factors. Immunoglobulin A (IgA) protease activity has been demonstrated in all tested ureaplasma strains representing 13 of the 14 serovars (UUR13 was not tested) [16,17]. IgA protease has been considered as one of the major factors contributing to the pathogenic potential of ureaplasmas [16,17]. It is expressed in bacterial pathogens especially when they are colonizing a mucosal surface [18]. This can provide them with an advantage in evasion of the host-defenses. It is interesting to note that commensal species of the genus *Neisseriae* do not express this enzyme [19]. Another potential pathogenicity factor is the release of ammonia through urea hydrolysis [10]. Ureaplasmas have also been reported to have phospholipase A1, A2 and C activities [20-23]. When an infection reaches the amnion or placenta, this phospholipase activity could lead to production of free arachidonic acid. This could activate the synthesis of prostaglandins and possibly induce labor prematurely. An intact humoral immune response appears to be important in limiting invasion and dissemination of ureaplasma beyond mucosal surfaces. This is demonstrated by their tendency to cause chronic respiratory infections and arthritis in persons with hypogammaglobulinemia, and to cause invasive disease in preterm neonates [10]. We sequenced the 14 ATCC UPA and UUR serovars as an effort to aid the development of serotyping methods and to enhance the study of the suggested differential pathogenicity [10] and ureaplasma biology. Based on these sequences real-time PCR genotyping assays were developed that detect the 14 ATCC serovars without cross- reactions [12]. Surprisingly, the application of these assays to 1,061 clinical isolates failed to correlate specific serovars with different clinical outcomes. Our inability to correlate patient disease outcomes with specific serovars was at least in part because a large fraction of those patient samples were classified as genetic hybrids. This result was based on our serotyping PCR assays. DNA sequencing of parts of some of the hybrid genomes showed that serotype specific markers were transferred horizontally among ureaplasmas [24]. Combining these findings with the comparative genome analysis of the 14 ureaplasma ATCC serovars has allowed us to better understand the potential mechanisms and reasons for these observations among clinical isolates. We report on genes that may contribute to the virulence of ureaplasmas, including the MBA and its putative mechanism of phase variation.

## Results and discussion

### Genome sequencing of 19 *U. Urealyticum* and *U. Parvum* strains

Subsequent to the publication and annotation of the complete genome of a clinical isolate of UPA3 by Glass and colleagues [25], sequencing of all 14 serovar type strains deposited in the ATCC was begun to study differences among them and examine them for virulence factors. The intent was to completely sequence the ATCC UPA3, which is the reference strain for UPA, and UUR8, which is the reference strain for UUR. The genomes of those serovars were completed along with UUR2 and UUR10. The sequencing coverage for each genome varied between 7X to 14.5X (Table 1). Genome sizes of UPA serovars were between 0.75–0.78 Mbp and of UUR serovars between 0.84–0.95 Mbp. We sequenced the genomes of four UUR clinical isolates that were negative for all of our serovar genotyping real-time PCR assays [26]. All of the isolates’ genomes had some minor genome rearrangements, regions that were deleted, and some regions that were inserted and are new for the urealyticum group when compared to the ATCC reference strains. Additional information for these regions can be found in the Additional file 1. Whether we can assign new serovar numbers to any of the unidentifiable isolates is a matter of clarifying the requirements for an ureaplasma to be considered a specific serovar.

**Table 1:** **Overview of *Ureaplasma urealyticum *and *Ureaplasma parvum *genomes**

**Serovar**	**ATCC**	**GenBankaccession**	**PFGE size (kbp)**	**Genome size (bp)**	**Contigs**	**ORFs**	**Hypothetical proteins**	**% GC**	**Sequence coverage**
**1**	27813	NZ_ABES00000000	760	753,674	8	604	212	25%	14.6X
**3**	27815	NC_010503	760	751,679	1	609	219	25%	10.2X
**3**	700970	NC_002162	Patient Isolate	751,719	1	614	154	25%	-
**6**	27818	NZ_AAZQ00000000	760	772,971	5	619	221	25%	11.4X
**14**	33697	NZ_ABER00000000	760	749,965	7	594	199	25%	14.5X
**2**	27814	NZ_ABFL00000000	880	861,061	1	664	248	26%	10.7X
**4**	27816	NZ_AAYO00000000	910	835,413	4	654	206	26%	7.0X
**5**	27817	NZ_AAZR00000000	1140	884,046	18	677	252	26%	8.5X
**7**	27819	NZ_AAYP00000000	880	875,530	4	660	246	26%	8.3X
**8**	27618	NZ_AAYN00000000	890	874,381	1	673	232	26%	9.9X
**9**	33175	NZ_AAYQ00000000	950	947,165	10	711	244	26%	8.6X
**10**	33699	NC_011374	890	874,478	1	657	232	26%	12.1X
**11**	33695	NZ_AAZS00000000	840	876,474	6	644	236	27%	10.0X
**12**	33696	NZ_AAZT00000000	870	873,466	2	650	234	25%	9.0X
**13**	33698	NZ_ABEV00000000	900	846,596	5	655	234	25%	11.1X
**2033**	unknown serovar	AJFX00000000	Patient Isolate	804,560	16	646	190	26%	39.0X
**2608**	unknown serovar	AJFY00000000	Patient Isolate	856,546	14	667	258	26%	60.0X
**4155**	unknown serovar	AJFZ00000000	Patient Isolate	858,890	18	684	225	26%	73.0X
**4318**	unknown serovar	AJGA00000000	Patient Isolate	844,630	16	662	214	26%	52.0X

### Gene content analysis

**Figure 1:**
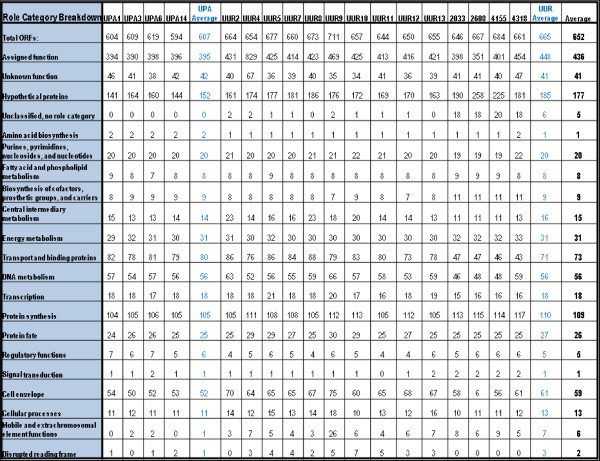
Role Category Breakdown of Genes.

All strains had the expected two rRNA operons and tRNA coding genes. A table of the tRNA species (Additional file 2: Figure S2) can be found in the supplementary materials. UPA serovars have an average of 608 genes, of which 201 encode hypothetical proteins on average, and UUR serovars have an average of 664 genes, of which 230 encode hypothetical proteins on average (Figure 1). The ureaplasma pan genome based on all 19 sequenced ureaplasma genomes contains 1020 protein coding genes of which 758 genes have orthologs in at least one other ureaplasma strain, and 515 genes are universally conserved among all 19 strains (ureaplasma core genome). The number of genes identified only in the genome of single serovars (singletons) is 262. The average number of singletons per genome is 14, however the range is wide (0 singletons in ATCC UPA3 and 68 in ATCC UUR9). Table 2 compares the pan genomes of different sets of ureaplasma species.

**Table 2:** **Pan genomes of different*Ureaplasma*species sets**

	**All 19 strains**	**14 ATCC serovars**	***U. urealyticum* (14 strains^a^)**	***U. parvum* (5 strains^b^)**
Pan genome	1020	971	938	688
Core genome	515	523	553	538
Singletons	262	246	216	77
Clusters of Orthologous Genes(COGs)	758	725	722	688

Pan genome represents the number of clusters of orthologous genes and singletons. Singletons are genes found only in one of the genomes. Clusters of Orthologous Genes (COGs) have genes orthologous among at least 2 genomes.

a) ATCC UUR2, UUR4, UUR5, UUR7-13, and the clinical isolates 2033, 2608, 4155, 4318.

b) ATCC UPA1, UPA3 (ATCC 27815), UPA3 (ATCC 700970), UPA6, UPA14.

**Figure 2:**
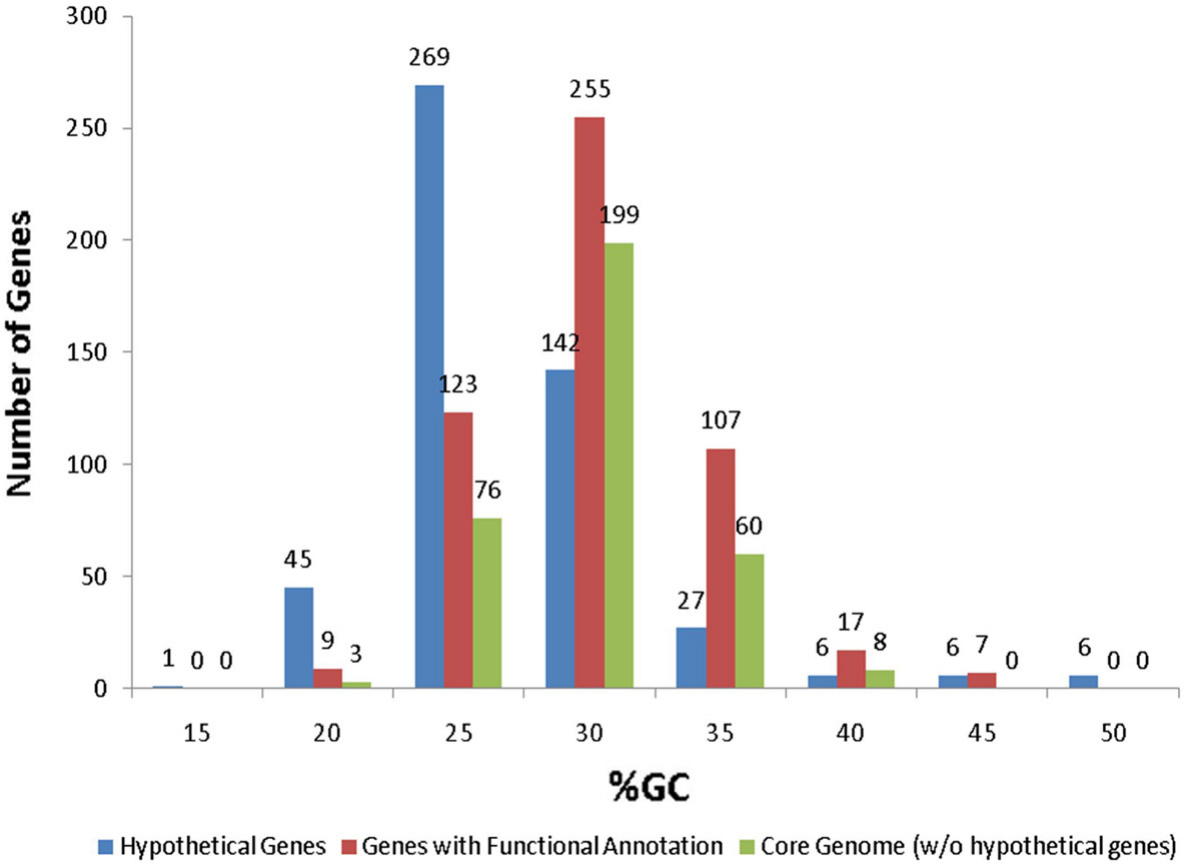
**Percent GC Distribution Among Genes of The Ureaplasma Pan Genome (19 Strains).** For genes that are present in two or more genomes the average %GC of the orthologs was used. Values were grouped in bins (example: bin 20 contains genes with %GC from 15 to 20%). %GC of singleton genes was also included in the histogram.

It has been suggested that genes that are not affected by the selective pressure on mycoplasmas gradually mutate at a faster rate than genes whose sequences are highly conserved to a higher AT content and eventually are lost [25]. Therefore, the %GC content may point out which genes are important for ureaplasmas or have recently been acquired horizontally. We evaluated the percent GC content of all genes across the 19 sequenced strains. Genes encoding hypothetical surface proteins conserved across all ureaplasma strains with high GC content may play an important role for ureaplasmas in processes like adherence to mammalian cells and colonization. An interactive excel table of the %CG values of all ureaplasma strains can be found in the Additional file 3: Comparative paper COGs tables.xls. A histogram of the distribution of %GC values of the ureaplasma pan genome shows that core genome genes with assigned function generally have a higher GC content than hypothetical genes (Figure 2). The median for the core genome was 27%GC, therefore genes with %GC higher than 27 are likely to be essential and/or acquired. The median for the hypothetical proteins was 24%GC. Considering that the ureaplasma genomes have an overall 25%GC content, it is likely that genes with GC content below 25% may be non-essential and on their way to be lost. The lowest GC content is of a hypothetical protein with only 13%GC content. The genomes of the 14 sequenced ATCC ureaplasma serovar strains showed extreme similarity between the two species and 14 serovars. The comparison of the finished genomes shows synteny on the gene level and not many rearrangements. We obtained percent difference values by whole genome comparison on the nucleotide level. The average intra-species percent difference was 0.62% with the least difference between UUR4 and UUR12 of only 0.06%, and the greatest difference between UUR9 and UUR13 of 1.27%. On the inter-species level the average percent difference was 9.5%, with the greatest difference between UPA1 and UUR9 of 10.2% (Table 3). As mentioned earlier, UUR serovars have about 118 Kbp (13.5%) larger genomes than UPA serovars. As a result UUR serovars have on average 58 genes more than UPA serovars.

**Table 3:** Serovar to serovar difference expressed in percent

	**1**	**3**	**6**	**14**	**2**	**4**	**5**	**7**	**8**	**9**	**10**	**11**	**12**	**13**
**1**		0.66	0.52	0.75	9.90	9.99	9.68	9.78	9.66	10.23	9.84	9.70	9.93	9.79
**3**	0.70		0.49	0.35	9.93	9.67	9.33	9.43	9.33	10.01	9.43	9.36	9.66	9.84
**6**	0.62	0.52		0.50	9.82	9.82	9.40	9.49	9.38	9.95	9.53	9.42	9.76	9.75
**14**	0.83	0.33	0.45		9.92	10.01	9.59	9.69	9.57	9.99	9.70	9.60	9.95	9.83
**2**	9.82	9.87	9.58	9.81		0.86	0.74	0.78	0.76	1.25	0.74	0.77	0.86	0.84
**4**	9.90	9.60	9.57	9.83	0.94		0.69	0.64	0.69	0.82	0.88	0.66	0.07	0.80
**5**	9.72	9.31	9.25	9.52	0.72	0.60		0.15	0.13	0.66	0.56	0.16	0.58	0.66
**7**	9.72	9.32	9.25	9.52	0.82	0.60	0.16		0.15	0.66	0.53	0.11	0.60	0.67
**8**	9.76	9.35	9.27	9.54	0.71	0.59	0.08	0.10		0.61	0.51	0.11	0.59	0.65
**9**	10.90	9.83	9.60	9.71	1.21	0.72	0.63	0.62	0.60		0.85	0.63	0.75	1.08
**10**	9.79	9.35	9.29	9.56	0.70	0.81	0.51	0.48	0.51	0.87		0.46	0.80	0.43
**11**	9.73	9.33	9.25	9.52	0.80	0.61	0.16	0.11	0.16	0.67	0.51		0.60	0.64
**12**	9.85	9.58	9.52	9.79	0.93	0.06	0.67	0.64	0.69	0.85	0.87	0.65		0.80
**13**	9.70	9.74	9.47	9.66	0.97	0.86	0.79	0.76	0.75	1.27	0.56	0.74	0.86	

The percent difference was obtained by whole genome comparison on the nucleotide level.

Fifty percent of these extra genes encode hypothetical proteins, the rest are spread among different functional categories (Figure 1). Table 4 shows the predicted genes present only in UUR serovars or only in UPA serovars. As it is seen in Figure 1, UUR had more genes encoding cell surface proteins, DNA restriction modification enzyme genes (see Additional file 3: Comparative paper COGs tables.xls) and remnants of transposons (truncated genes or genes with unverified frameshifts). Furthermore, there are subtle differences in the predicted activities of proteins encoded by various reductase genes among serovars, which may facilitate unequal resistance of different ureaplasmas to oxidative stress during colonization and infection.

**Table 4:** Number of Clusters of Orthologous Genes (COGs) per functional category present only in UUR or UPA serovars

***Ureaplasma urealyticum***	
**Present in at least two UUR genomes (none in UPA)**	**#COGs**
hypothetical protein	83
putative lipoprotein	8
multiple banded antigen	7
putative membrane protein	4
transposase	4
DNA primase	3
DNA topoisomerase IV, B subunit	3
site-specific recombinase	3
restriction-modification enzyme subunit	2
AAA domain/DeoR HTH domain protein	1
AAA family ATPase	1
ABC transported MDR-type, ATPase component	1
chromosome partition protein Smc	1
divergent AAA domain family	1
ferrichrome ABC transporter, ATP-binding	1
putative phage head-tail adaptor	1
relaxase	1
sigma-70, region 4 family	1
superfamily II DNA and RNA helicase	1
TolA homolog	1
TraG/TraD family	1
viral A-type inclusion protein, putative	1
***Ureaplasma parvum***	
**Present in at least two UPA genomes (none in UUR)**	**#COGs**
hypothetical protein	18
type I restriction modification enzyme protein	3
integrase-recombinase protein	2
putative lipoprotein	2
divergent AAA domain family	1
nucleoside 2-deoxyribosyltransferase sup	1

### Ureaplasma phylogenetic tree

Constructing an accurate phylogenetic tree that resolves the relationship of ureaplasma serovars has been difficult due to the extreme similarity of these organisms on the genome level. Several methodologies exist for the construction of phylogenetic trees: single gene trees, trees based on concatenated gene sequences, gene content trees, and gene order trees. Phylogenetic trees based on single genes are unlikely to provide an accurate lineage of the serovars because of horizontal gene transfer among ureaplasmas. We find extensive horizontal gene transfer among clinical isolates relative to the 14 ATCC type strains [26]. Another challenge of building intra-species phylogenetic trees based on a single gene is that the primary nucleotide sequences of the genes conserved among all ureaplasma serovars/strains have such a high percentage of identity that there are not enough informative positions in the multiple sequence alignment to provide a resolution capability with high confidence. A gene content tree is based on a multiple sequence alignment in which each sequence (line) represents the genome of a strain and each position (column) in the multiple sequence alignment signifies the presence or absence of a gene in the strain. Therefore, such a tree has a binary nature (presence = 1, absence = 0). The pan genome of ureaplasmas generates a relatively short multiple sequence alignment: 1020 positions for 1020 genes in the pan genome. Therefore, a gene content tree of ureaplasma strains does not have the fine resolution capability of a phylogenetic tree based on nucleotide sequences. This can be noted in the low bootstrap values of the deep nodes of the gene content tree based on the pan genome (Additional file 4: Table S1). We did not attempt to construct a gene order tree, because the majority of the genomes are in multiple pieces, thus making it hard to judge the gene order in these genomes.

**Figure 3:**
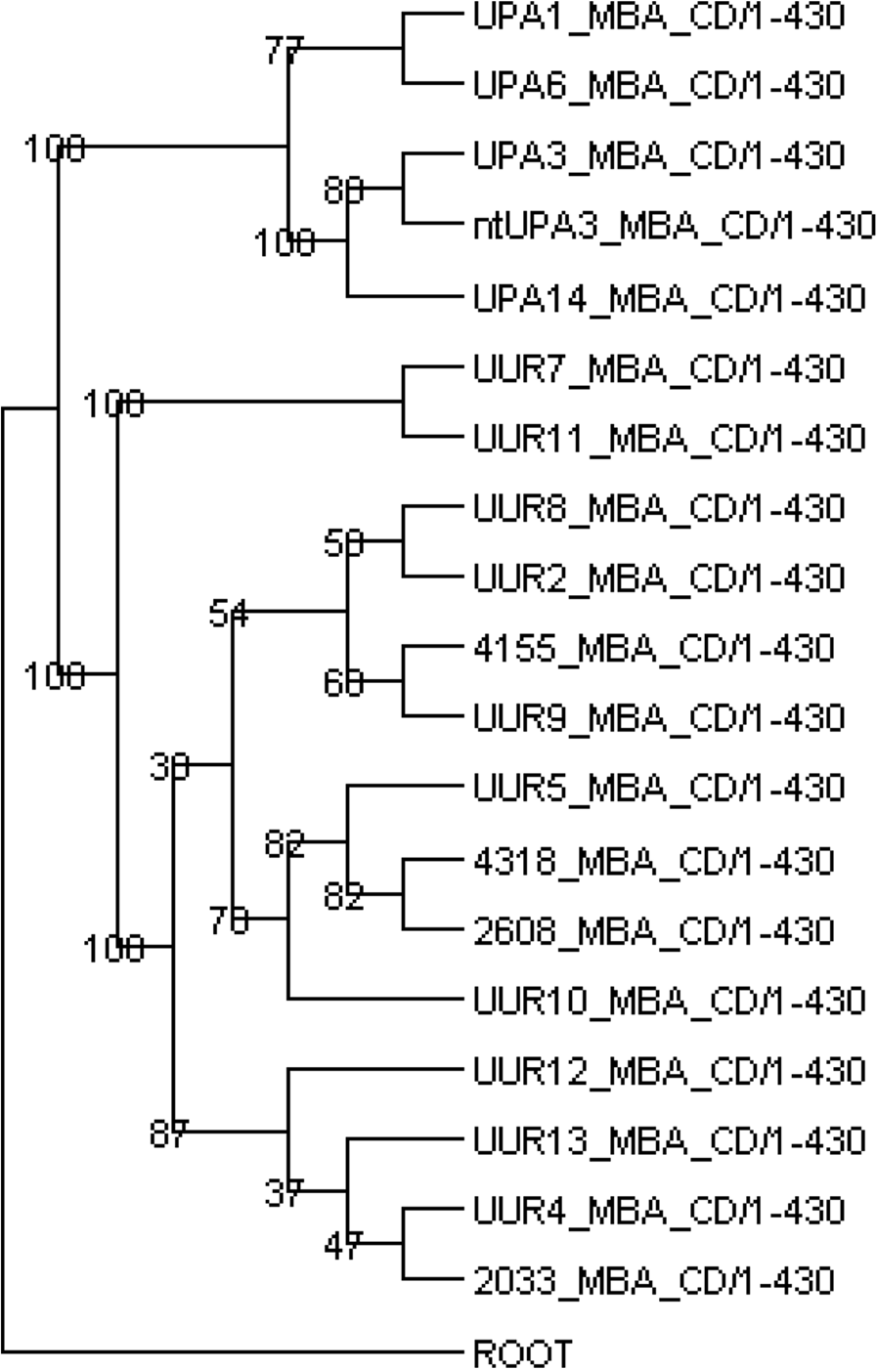
**MBA Based Phylogenetic Tree of 19 Ureaplasmas.** The tree is based on the nucleotide sequence of the conserved domain of the *mba* (1–430 nt).

**Figure 4:**
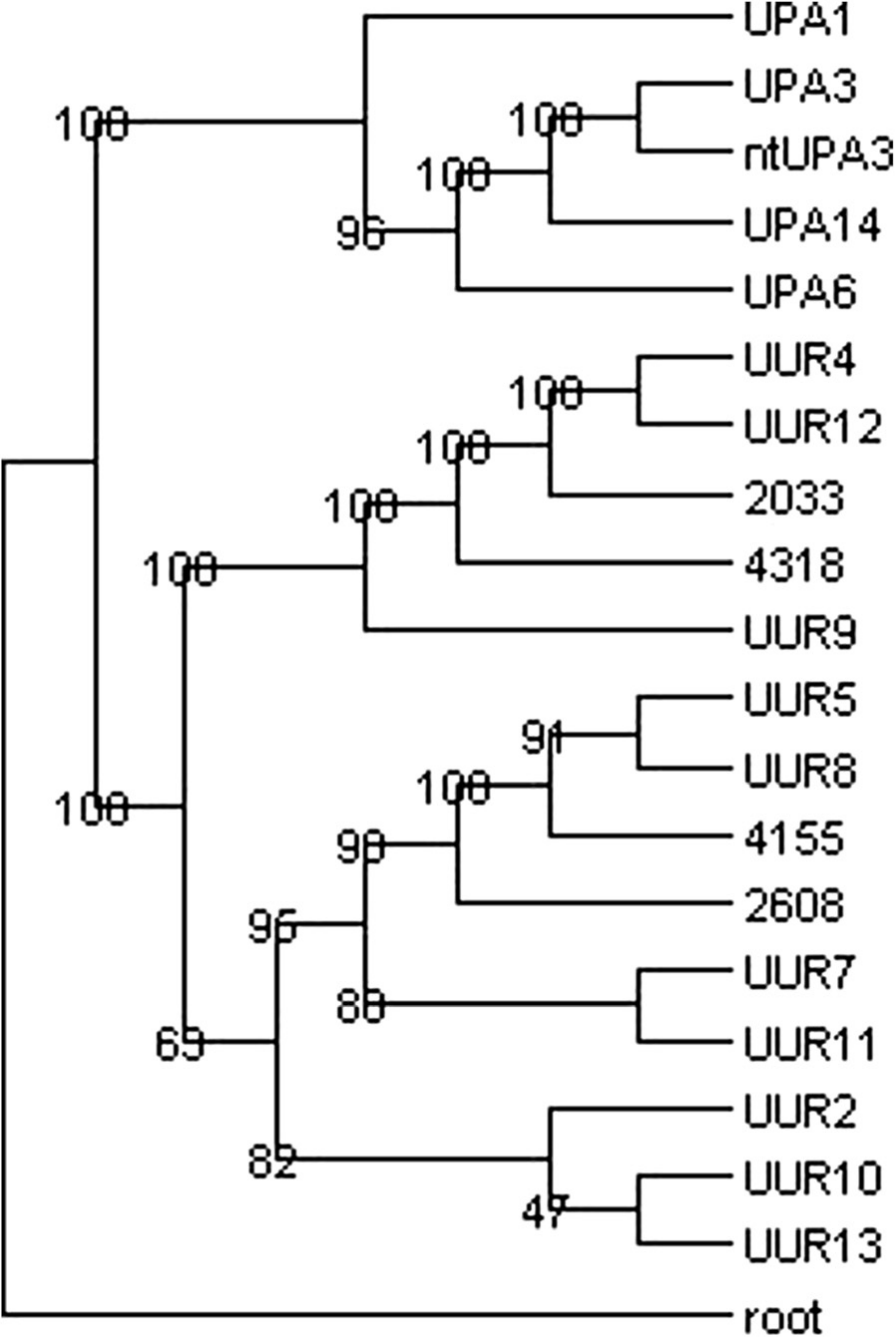
**Phylogenetic Tree of 19 Ureaplasma Strains Based on 82 Housekeeping Genes.** ATCC type strains are labeled with tree letters (species) followed by a number (serovar). UUR = *Ureaplasma urealyticum*; UPA = *Ureaplasma parvum*; ntUPA3 = clinical isolate sequenced in 2000; 2033, 2608, 4155, and 4318 are clinical isolates of *Ureaplasma urealyticum* that cannot be serotyped. The tree is based on the concatenated alignment of 82 housekeeping genes 16 tRNA ligase genes, 12 DNA and RNA polymerase genes, 47 ribosomal protein genes, and the 7 urease subunit genes). The non- informative positions were removed from the alignments. The removal of the non- nformative positions increased the bootstrap values.

Phylogenetic trees of ureaplasmas have been published previously, showing clear separation of the *parvum* and *urealyticum* species [27,28]. The conserved domain of the *mba* genes has been used to generate a phylogenetic tree to resolve the relationship of serovars [5,29]. We reconstructed the *mba* conserved domain tree using the first 430 nucleotides of the *mba* gene of all 19 strains (Figure 3). We also present a phylogenetic tree (Figure 4) based on the information of the nucleotide sequence of 82 housekeeping genes forming four groups: 1) 16 tRNA ligase genes 2) 12 RNA and DNA polymerase genes, 3) 47 ribosomal protein genes, and 4) 7 ureases. The clades of the multigene tree are very similar to the clades of the previously published *mba* based tree; however, the deep nodes of the two trees show some differences. These differences may be due to differences in the gene acquisition events that are averaged in a phylogenetic tree based on multiple genes versus a single gene tree. Similar differences in the deep tree nodes can be seen in the phylogenetic trees resulting from the concatenated alignments of the genes of each of the four groups and the trees resulting from different combinations of the groups (Additional file 2: Figures S2–S4). However, as more genes are used to construct the trees, the clade and node structure of the trees becomes more consistent.

### Recombination and integration of DNA

All ureaplasma serovars contained one or more integrase-recombinase genes and some serovars contained transposases, or remnants of transposases, and some phage related proteins. Most of the recombinases were site-specific tyrosine recombinases, which are present also in other mycoplasmas and firmicutes. The highest number and variety of such genes was observed in serovar 2, and in general, UUR serovars had higher number of these genes than UPA serovars. However, insertion events represented only a small portion of the average 118 Kbp difference between the two species. A gene encoding a site-specific integrase-recombinase was adjacent to the phase variable locus of the MBA in 12 of the 14 serovars. This recombinase was likely involved in the rearrangements of the *mba* locus resulting in the variation of the C-terminal of this surface antigen. The presence of transposases suggested that foreign mobile DNA elements have been inserted in the genomes of ureaplasma serovars. Some of the transposases have truncations or unverified frameshifts indicating that the mobile element that they were part of was most likely no longer mobile. It was no surprise to find transposon related genes in serovar 9, which had acquired tetracycline resistance. The *tetM* gene was identified as part of a Tn916 transposon, based on the genes around it. Although tetracycline-resistant ureaplasma were probably less frequent when serovar 9 was isolated, now they comprise 25–35% of all patient isolates. A report covering the years 2000–2004 from several states in the USA showed that 45% of unique clinical isolates of *Ureaplasma* spp. contain *tetM* and are tetracycline-resistant [10]. Further evidence of genome integrated transposons were some of the site-specific recombinases found in the genomes: TnpX, required for the excision of Tn4451 [10] and TndX, which was the first member of the large-resolvase subgroup of the resolvase/invertase family of site-specific recombinase shown to be able to mediate the insertion and excision of a conjugative transposon, more specifically Tn5397 [30].

A TraG/D family protein was recognized in serovars 9 and 13 (UUR9_0186 [GenBank: ZP_03079565] and UUR13_0031 [GenBank: ZP_02932006]). The TraG/D (transport) family genes aid the transfer of DNA from the plasmid into the host bacterial chromosome [31,32], mediate the interactions between the DNA processing (Dtr) and mating pair formation (Mpf) systems during conjugation. Another suggestion for the capacity of horizontal gene transfer in at least some serovars is the presence of relaxases/mobilization proteins (UUR9_0148 [GenBank: ZP_03079581] and UUR13_0045 [GenBank: ZP_02696018]). Such proteins are required for the horizontal transfer of genetic information contained on plasmids that occurs during bacterial conjugation [33]. Aligning the genomes of the 14 ATCC ureaplasma genomes made evident two major insertion events. The first one was consistent with a transposon insertion, due to the repeat of some host sequence on both sides of the inserted region. At the time of insertion a short part of the 3′ end of the *ruvB* was duplicated, so that the insertion was located between the full length *ruvB* gene and its short duplication. The insertion has been inherited by UPA1, 3, and 14 from a common ancestor. Some of the genes present in this insertion had orthologs in UUR serovars. The inserted DNA fragment was 11,822 bp long in UPA3 and 14, and 12293 bp in UPA1. It contained 8 genes, which encoded 6 hypothetical proteins, one hypothetical protein containing a subtilase domain, and one Type I specificity subunit restriction protein. The second insertion was present in 9 of the 14 serovars (UPA3, and 6, UUR4, 5, 7, 8, 10, 11, and 12) and had a size of about 20 Kb.

Based on the fact that there were three phage genes in the insert, we believe that this event is due to a phage insertion into the genomes. The first gene of the insertion encodes an integrase-recombinase protein that contains a phage integrase domain (UPA3_0153 [GenBank: YP_001752228]). A phage recombination protein Bet (UPA3_0162 [GenBank: YP_001752237] is located further downstream of the integrase and the final gene in the insert is a phage terminase, large subunit, of the pbsx family (UPA3_0176 [GenBank: YP_001752251]. The rest of the genes are hypothetical proteins, however some of them have one or more transmembrane domains and/or signal peptides, suggesting that they may play a role on the surface of the ureaplasma cell. It is important to note that the same exact insertion regions have been identified through a comparative genomic microarray analysis of 10 UPA clinical strains [34]. In this comparative genome microarray study these two insertions were present in some isolates of the same serovar and absent in other isolates of the same serovar. The authors suggest the phage insertion might be a putative pathogenicity island. Although the C + G content of the insertion is less than 1% higher than the rest of the genome, Momynaliev and colleagues [34] found that GCGC and CGCG tetranucleotides, that are present in ureaplasma DNA fragments, were missing in the inserted DNA fragment, thus providing another clue of the foreign character of the inserted DNA fragment.

Examining the putative restriction-modification (RM) genes in the 14 serovars (Additional file 3: Table S3) suggests that, although each serovar has from six to twelve RM genes, most RM systems are incomplete. Serovars 3, 5, 7, 8, 10, and 11 may have a complete type III RM system, serovar 9 may have a complete type I and type II RM system, whereas serovars 1, 14, 2, 12, and 13 appear to have only remnants of RM systems. It appears that all serovars have orthologs of the *hsd* specificity and/or methylation subunits belonging to the type I RM system. In all serovars, except UPA3 and UPA14, these orthologs are most similar to the *hsd* genes of *Mycoplasma pulmonis,* which are phase variable [35-37]. We found evidence of rearrangement of a pair of *hsdS* genes in the unfinished genome of UPA1. On the UPA1 main contig (gcontig_1106430400171, 734075nt) the two genes were adjacent and oriented in opposite directions, whereas on a small contig (gcontig_1106430400162, 2207nt), which contained only these two genes, the genes are adjacent and oriented in the same direction. Further investigation is necessary to determine whether these RM genes indeed phase- vary and what is the mechanism for their phase-variation. RM systems are used in general by organisms to protect themselves from foreign DNA like viruses. Although phages that infect ureaplasmas have not been reported, the existence of these RM systems, as well as the presence of either intact or remnants of RM systems in the other urogenital mycoplasmas *M. genitalium* and *M. hominis* suggests that there are phages that infect these obligate parasites. In organisms like *Chlamydia* spp., which are obligate intracellular parasites and have no identifiable infecting viruses, there are no functional RM systems [38].

### Potential pathogenicity genes

#### Phospholipase C, A_1_, A_2_

Phospholipase C, A_1_, and A_2_ (PLC, PLA1, PLA2) activity was reported in Ureaplasma serovars 3, 4, and 8 by DeSilva and Quinn [20,21,23]. It is important to note that the assay used by DeSilva measures combined activity of PLC and phospholipase D (PLD) because both cleavage products are in the soluble fraction and the radioactively labeled hydrogen would be found in both cleavage products [39]. PLC activity has been reported in *Ureaplasma diversum* cells as well, and has been suggested to play a role in ureaplasma invasion in mammalian cells [40]. However, the detection method used the artificial substrate p-nitrophenylphosphorylcholine (p-NPPC), which can be hydrolyzed by several other enzymes that can hydrolyze phosphate esters, including PLD [41]. All 14 ATCC ureaplasma serovar genomes and the genome of the previously sequenced clinical isolate of UPA3 were extensively evaluated for the presence of PLC, PLA1, and PLA2 genes. No genes showed significant similarity to known sequences of PLC, PLA1, or PLA2 in any of the genomes. HMMs developed for known PLC, PLA1, and PLA2 did not detect any ureaplasma genes with significant similarity. This suggested that ureaplasma may encode phospholipases that are either very degenerate or have evolved separately from known phospholipases as previously suggested by Glass et al. [25], or that no phospholipase genes are present in *Ureaplasma* spp. It is interesting to note that a PLD domain containing protein was easily identified. In all serovars this protein is annotated as cardiolipin synthase (UPA3_0627 [GenBank YP_001752673]).

We used two PLC assays to test ureaplasmas for PLC activity: Invitrogen’s Amplex® Red Phosphatidylcholine-Specific Phospholipase C Assay Kit, which detects also PLD activity, and the original PLC assay published by DeSilva and Quinn. We were not able to detect PLC or PLD activity in ureaplasma cultures of serovars 3 and 8. Our attempts to repeat De Silva and Quinn’s PLC assay using L-a-dipalmitoylphosphatidylcholine - (choline-methyl-3 H) with UPA3 and UUR8 cultures grown to exponential phase and processed to collect the cell membranes and cleared cell lysates as described in their original publications [20,21,23] failed to replicate the specific activity levels they reported in ureaplasma cultures. Because we were not able to find PLC, either computationally or experimentally, we believe that this gene is not present in ureaplasmas. However, a study done by Park et al. suggests implication of PLD in the signaling cascade that activates COX-2, leading to production of prostaglandins and initiation of labor [42]. Since all ureaplasma serovars and the four sequenced clinical isolates contain a gene with PLD domains, a future functional characterization of this gene would be of interest. We have not been able to find computationally the genes encoding PLA1 and PLA2 in ureaplasmas.

#### IgA Protease

In the mammalian immune system, a primary defense mechanism at mucosal surfaces is the secretion of immunoglobulin A (IgA) antibodies. Destruction of IgA antibodies by IgA specific protease allows evasion of the host defense mechanism. In *Neisseria gonorrhoeae* the IgA protease doubles as a LAMP-1 protease to allow it to prevent fusion of the phagosome with the lysosome [43]. IgA protease activity was demonstrated in ureaplasma serovars [16,17]. All sequenced human ureaplasma genomes were evaluated for IgA protease genes with the same methods as the phospholipases gene search. We could not computationally identify an IgA protease gene.

#### Nucleases

Nucleases have been reported as potential pathogenicity factors in other organisms as well [44]. Ureaplasmas belong to a group of organisms that import nucleotides for DNA and RNA synthesis. Therefore it is likely that they have secreted or surface bound nucleases that may also play a role in pathogenicity. We identified 15 potential nucleases, of which two had a predicted signal peptide, and thus are likely to be secreted or surface bound. These nucleases may be an interesting target for further studies of their potential involvement in pathogenicity.

#### Putative O-sialoglycoprotein peptidase

Eleven of the 14 ureaplasma serovars contained a gene annotated as an O-sialoglycoprotein endopeptidase (UPA3_0428 [GenBank: ACA33260]). UUR serovars 2, 8, and 10 did not contain an ortholog of this gene. Because all three of these genomes are complete (no gaps in the genome sequence), we can be sure the gene is absent. This enzyme has been shown to cleave human erythrocyte glycophorin A in other bacteria [45]. The same study showed that the specificity of this peptidase is limited to O- glycosylated membrane glycoproteins, and it cannot cleave N-glycosylated proteins. Abdullah et al. [45] suggest that the potential targets of this enzyme in the host are sialoglycoproteins of the mucosal epithelial cells or on the cell surfaces of macrophages. In fact the O-sialoglycoprotein peptidase of *Mannheimia haemolytica* cleaves from the surface of the human cell line KGla the CD43-leukosialin and other human O- sialoprotein antigens like the progenitor cell-restricted antigen CD34, the hyaluronate receptor CD44, and the leukocyte common antigen tyrosine phosphatase CD45 class of molecules [45]. If the ureaplasma putative O-sialoglycoprotein peptidase is capable of cleaving such targets, this could be a mechanism for evasion of the host immune system, colonization of the host, and eventually establishment of an infection. In *M. haemolytica* isolates the presence of this gene is associated with the capacity of the bacteria to cause pneumonia in calves [45].

#### Macrophage infection mutant protein, MimD

UUR2 contained a gene annotated *mimD* (UUR2_0526 [GenBank: ZP_03771352]) standing for macrophage interaction mutant D. *Mycobacterium marinum* is a fish, amphibian, and human pathogen that may be able to survive and replicate in macrophages. A study of macrophage infection *D. marinum* mutants identified a mutation in a hypothetical protein that resulted in this phenotype [46]. The exact function of this gene in interactions with macrophages is not yet defined; however the ureaplasma annotated *mimD* gene (183 aa) had 40% identity and 68% similarity over 179 aa long alignment with the *M. marinum mimD* gene (731 aa). Further characterization of MimD in other systems and possibly ureaplasma would be interesting.

#### Resisting hostile environment

Bacteria are known to produce substances that give them competitive advantages over other bacteria in their environment. Some of these substances are bacteriocins (like mutacin produced by *Streptococcus mutans*) and H_2_O_2_ to inhibit the growth of other bacteria [47]. UUR13 has two of the three suggested genes involved in immunity to mutacin, *mutE* and *mutG*[48]. A gene encoding a peroxidase in the ancestral ureaplasma has diverged to encode a likely glutathione peroxidase gene [GenBank: ACA33207.1] in all UPA serovars and a likely peroxiredoxin [GenBank: ZP_03772062] in all the UUR serovars. These genes could play a role in resisting oxidative stresses and bacteriocins produced by the rest of the bacteria on the mucosal surfaces they occupy. We detected a thioredoxin reductase system in all 19 genomes [GenBank: ACA33034 and NP_078428]. The thioredoxin reductase system has been described previously in mycoplasmas and has been suggested to function as a detoxifying system to protect the organism from self generated reactive oxygen compounds [49]. The presence or absence of such genes in an individual ureaplasma strain may contribute to the difference of pathogenic potential of the strain.

#### Multiple Banded Antigen (MBA) Superfamily

The original classification of ureaplasma isolates into distinct serovars was largely based on differences in the major ureaplasma surface antigen called the multiple banded antigen (MBA) (8–10, 12). MBA consists of an N-terminal conserved domain and a C-terminal variable domain. The conserved domain contains a signal peptide, lipoprotein attachment site, and one transmembrane domain. While the conserved *mba* domains for all 14 serovars had been sequenced previously, for most serovars sequencing of the variable domain, which was thought to be serovar specific, was only partial [15,50,51]. Our whole genome data confirmed that variable regions usually consist of tandem repeating sequence/units (TRU). Only in UUR13 is the conserved domain attached to a variable domain that does not contain any tandem repeats. The same variable domain is found also in UUR12 and UUR4; however it is not attached to the conserved domain of the *mba* in these serovars*.* The MBA is recognized by the Toll-like receptors 1, 2, and 6, and is capable of inducing the cytokine, NF-κB and antibody production [52]. It is conceivable that ureaplasmas would have evolved strategies to vary the MBA in order to evade this response. Ureaplasma isolates can vary the number of the tandem repeats of their *mba* gene in response to challenge with antibodies presumably by slipped strand mutagenesis [53]. Furthermore, *mba* can phase vary with neighboring genes, and UPA3 was recently shown to produce a chimeric genes though phase variation by fusing the N- terminal part of the *mba* paralog UU172 [GenBank: CBI70486] to its neighboring gene UU171 [GenBank: NP_078003] and by fusing the N-terminal part of UU375 [GenBank: NP_078209.1] to its neighboring gene UU376 [GenBank: NP_078210.1] [54,55]. These findings suggest that *mba* and some *mba* paralogous genes might be involved in strategies for evading the host immune system employed by ureaplasmas.

**Figure 5:**
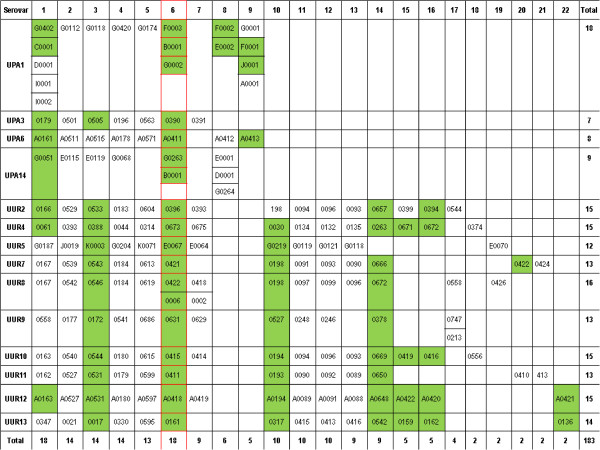
**Clusters of Orthologous Genes Potentially Involved in the MBA Phase Variable System of Ureaplasmas. ** This table contains the NCBI locus tags for genes potentially involved in the MBA phase variable system. To form the NCBI locus tag add the serovar id and underscore before the gene number: UPA1_G0402; UUR12_A0163. Genes with tandem repeats are highlighted in green. A red box is drawn around the 4MBA genes expressed in ATCC type strains.

**Figure 6:**
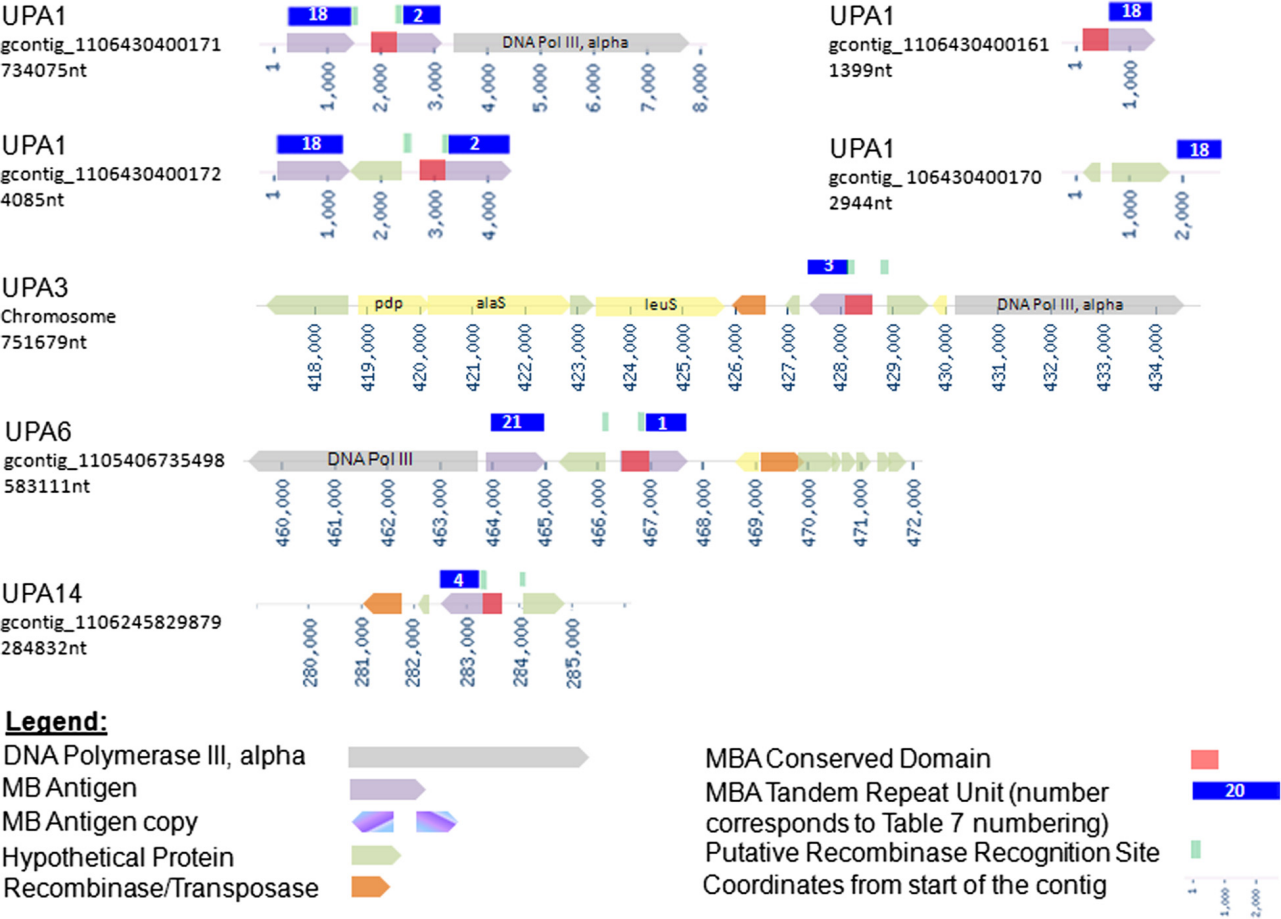
***Ureaplasma parvum *Multiple Banded Antigen Locus.**

**Figure 7:**
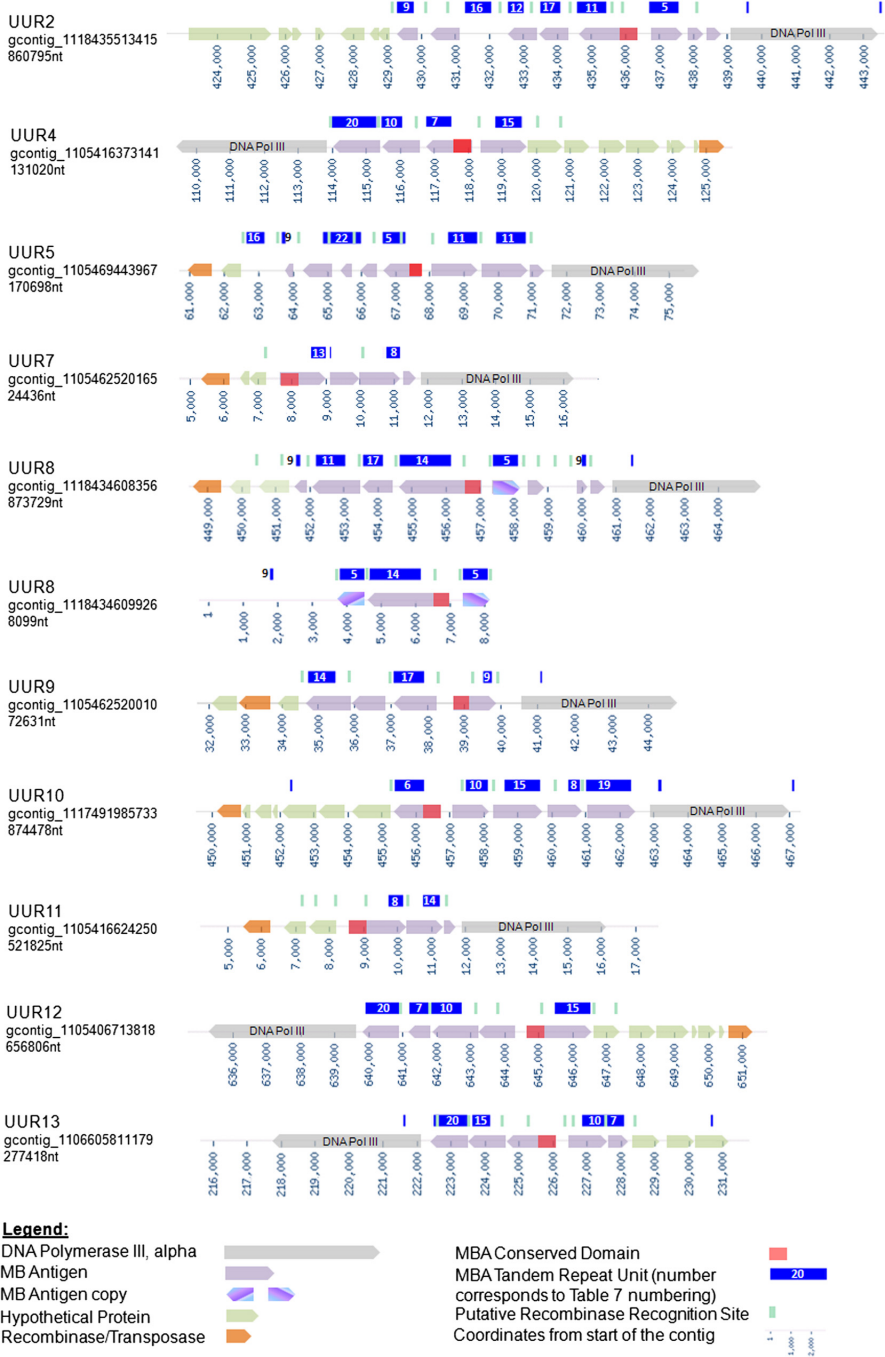
***Ureaplasma urealyticum *Multiple Banded Antigen Locus.**

One of the surprises of our whole genome analysis and comparison of the 14 ATCC serovars showed the *mba* genes to be part of a large complex gene superfamily comprising 183 UPA and UUR genes and 22 subfamilies (Figure 5). There were a limited number of unique variable domains as shown in Table 5. We found that all UUR serovars and UPA1 and 6 had more than one tandem repeating unit type in their *mba* locus. Although some of the TRUs in the loci have not yet been observed to be attached to the conserved domain of the *mba*, they are surrounded by inverted repeats that contain a putative recombinase recognition site. This suggested that these TRUs were involved with the *mba* and contributed to surface antigen variation. We consider genes without tandem repeats that are in the *mba* locus and have the putative recombination recognition site to be part of the MBA superfamily. The UPA serovars had a simpler MBA phase variation systems than the UUR serovars: the UPA conserved domain was surrounded by inverted single base pair repeats, containing the 25 base pair putative recombinase recognition site (Figures 6 and 7). The inverted repeats and a site-specific recombinase were potentially involved in inverting the orientation of the transcriptional promoter and conserved domain in order for expression to occur with one or the other TRU. A list of all genes encoding potential recombinases or transposases is provided in the Additional file 5: 19UU_Recombinases.xls. In most serovars a recombinase or a transposase is located in close proximity to the *mba* locus. Experimental evidence is needed to determine which recombinase is responsible for the rearrangement of the locus. It is interesting to note that one TRU was short and had a high copy number (18 nt - UPA1, 12 nt - UPA6, repeated >30X) and the other one was long and had a low copy number (327 nt -UPA1, 336 nt - UPA6, repeated <5X). Rearrangements of the *mba* locus were evident in the smaller contigs of unfinished serovar genomes (Figures 6 and 7). UPA1 genome sequencing data clearly shows a sub-population in which the conserved domain of the *mba* is attached to the alternative TRU ([GenBank: NZ_ABES01000008] -gcontig_1106430400161, [GenBank: NZ_ABES01000003] - gcontig_106430400170; Figure 6 & Table 5) and another subpopulation in which another gene is present between the two TRUs ([GenBank: NZ_ABES01000002] - gcontig_1106430400172). The high repeat number of the *mba* TRUs, and the existence of a subpopulation in the culture being sequenced that has a rearrangement of the *mba* locus, represent an ambiguity for the assembly software, resulting in the generation of smaller alternative contigs that cannot be assembled into the chromosome. The alternative 327 nt *mba* TRU of UPA1 is on a 1399 nt long contig [GenBank: NZ_ABES01000008] that contains only this gene, and it ends truncating the 327 nt TRU at only 2.3 repeats compared to 4 repeats on the main contig. Furthermore, comparing the two variations of the *mba* locus makes evident the break-points where the flip of the conserved domain occurred. This coincides with the sites of the inverted repeats suspected to be part of the mechanism for MBA phase-variation. This represents sequencing evidence that this serovar could express both variations of the MBA at different times.

**Table 5:** **Tandem Repeating Units (TRUs) identified in the*mba*locus**

	**Name**	**Period size (bp)**	**Copy # in sequenced ATCC**	**Serovars**	**Thought to be unique for serovar**	**Conserved domain attached in serovar (clinical isolate)**	**Clinical Isolates of UU; unknown serovar**
1	***mba12bp***	12	60.8	6	6	6	-
2	***mba18bp.1***	18	36.7–53.7	1	1	1	-
3	***mba18bp.2***	18	40.6	3	3	3	-
4	***mba21bp***	21	29.5–32.0	14	14	14	-
5	***mba24bp.1***	24	20.2–33.5	2,5,8	5	5 (2608, 4318)	2608, 4318, 4155
6	***mba24bp.2***	24	34.6	10	10	10	-
7	***mba30bp***	30	17.2–26.2	4,12,13	4	4 (2033)	2033
8	***mba42bp***	42	7.6–11.6	7,10,11	11	11	-
9	***mba45bp***	45	2.0–10.0	2,5,8,9	9	9	4155
10	***mba213bp.1***	213	3.0–4.0	4,10,12,13	-	-	2033
11	***mba213bp.2***	213	2.8–3.9	2,5,8	2	2	4155
12	***mba213bp.3***	213	1.9	2	-	-	-
13	***mba231***	231	2.8–3.9	7	7	7	-
14	***mba252bp.1***	252	1.9–5.9	8,9,11	8	8	4155
15	***mba252bp.2***	252	2.1–4.1	4,10,12,13	12	12	-
16	***mba252bp.3***	252	2.0–3.0	2,5	-	-	-
17	***mba276bp***	276	2.0–3.8	2,8,9	-	(4155)	2608, 4318
18	***mba327bp***	327	2.3–4.0	1	-	1	-
19	***mba330bp***	330	4	10	-	-	2608
20	***mba333bp***	333	3.0–4.0	4,12,13	-	-	2033, 4318
21	***mba336bp***	336	2.9	6	-	-	-
22	***mba579bp***	579	1.9	5	-	-	-

The name of each TRU consists of the *mba* gene name followed by the period size (bp) of the repeating unit. Different sequences of the same period size are marked by “.” and a version number (ex. *mba*18.1 and *mba*18.2). Observed minimum and maximum copy number of the TRU is shown in the third column. Column 6 shows the serovar in which the conserved domain was associated with each TRU. Note that the conserved region of the UPA1 *mba* was found linked to two different TRUs (highlighted).

**Figure 8:**
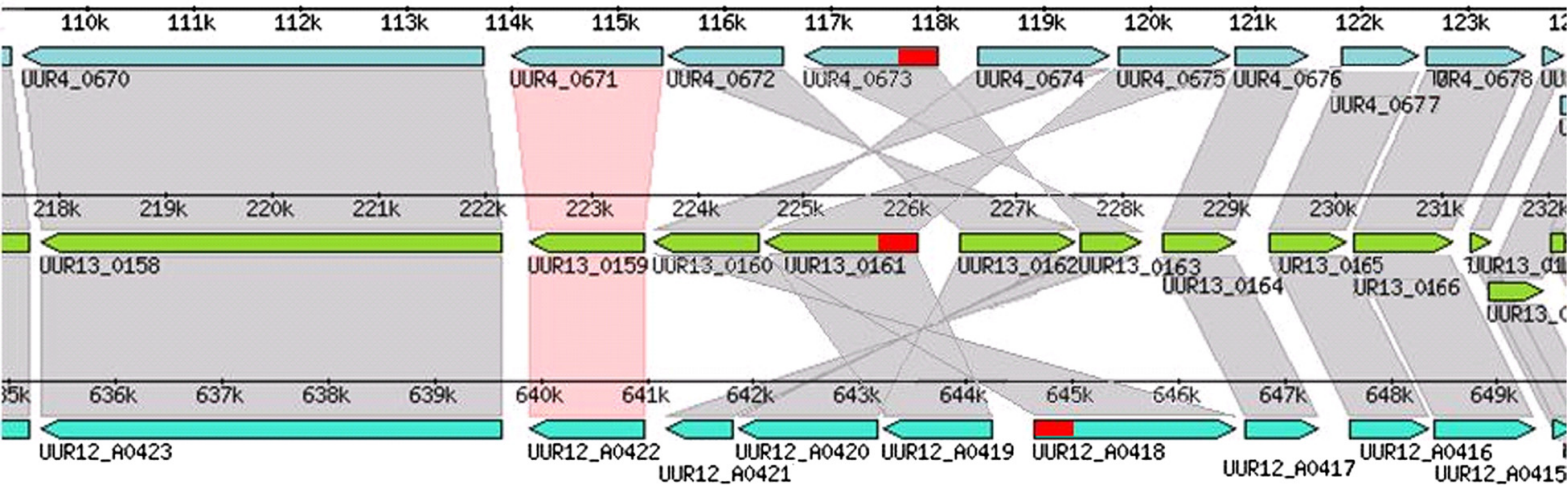
**The MBA Locus in UUR4, UUR12, and UUR13.** Genes in each genome are represented as directional blue or green boxes. Orthologous gene clusters (COGs) are represented by gray or pink bands spanning across the tree genomes. The COG with a pink band represents the first *mba* gene in the MBA locus. The locus includes the next 4 genes following the gene in the pink labeled COG (all tree genome have 5 *mba* genes each). The conserved domain of the *mba* is marked by a red box. Rearrangements of the genes are visible by following the twisting of the connecting bands.

All UUR serovars have more than two TRUs in close proximity to each other. Serovars UUR7 and UUR11 have only 2 TRUs each, whereas UUR2 and UUR5 have 6 TRUs each, which is the maximum number of TRUs observed. The largest *mba* loci are around 10 KB and have 6 TRUs and some non-TRU *mba* genes. Each *mba* locus contains only one conserved domain. The loci are always located adjacent to the DNA pol III alpha subunit (except UPA14) and on the other side of the loci there is a putative Xer-C site- specific recombinase. Next to each TRU there is a putative 25 nt recombinase recognition sequence [ACTTT(T/C)TCT(G/C)TTTGATAATT(C/A)AAAT]. The same recognition site is located next to some non-TRU genes in the loci, therefore making them likely to be involved in this phase variable superfamily. Furthermore, serovar 13 has a non-TRU variable domain fused to the conserved domain of the *mba*, confirming that the variable unit does not necessarily require tandem repeats. An interesting observation is that UUR4, 12 and 13 have the same *mba* locus composition in 3 different rearrangements (Figure 8). Most TRUs were found to be present in more than one serovar. By carefully analyzing small contigs in unfinished ureaplasma genomes, we identified variations of the *mba* loci. For example, on a small contig of UUR8 gcontig_1118434609926 [GenBank: NZ_AAYN02000001] we saw a partial *mba* locus arranged alternatively by duplicating one of the TRUs in the locus. Examining the sequencing and assembly data of such contigs confirms that these contigs are not misassembled, but rather represent a subpopulation of the sequenced culture. The proposed mechanism for variation of the ureaplasma *mba* locus resembles the previously reported variable loci of *Mycoplasma bovis*: *vsp*, *Mycoplasma pulmonis: vsa* and *Mycoplasma agalactiae: vpma*[56]. The involvement of a site-specific Xer-like recombinase and inverted repeats was experimentally proven for the *M. pulmonis vsa* locus [57] and the *vpma* locus of *M. agalactiae*[58]*,* and suggested for the phase variation of the *vsp* locus in *M. bovis*[56]*.* We believe that a Xer-like recombinase is likely to be involved in the phase variation of the *mba* locus of *Ureaplasma* spp and a putative recombinase recognition site has been determined. The *mba* locus resembles the *M. pulmonis vsa* locus in that it has only one promoter and one conserved domain per *mba* locus, which needs to be moved in front of a variable domain to make a functional surface MBA.

Examination of the *mba* loci of the four sequenced UUR clinical isolates that cannot be assigned to a serovar shows that the *mba* conserved domain is UUR specific. Due to the repetitive nature of the *mba* TRUs the loci are broken into multiple contigs, making it impossible to determine the exact order of the genes in the *mba* loci without further sequencing. Isolate 2033 had 4 identifiable TRUs (*mba*333bp, *mba*213bp.1, *mba*30bp and the non-tandemly repeating unit of UUR13; see Table 5). Of these, *mba*30bp was found attached to the conserved domain of the MBA and is the equivalent of the active TRU in UUR4. The same TRU was also present in the mba loci of UUR12 and UUR13. Isolate 2608 contained 3 identifiable TRUs (*mba*24bp.1, *mba*267bp, and *mba*330bp). The conserved domain was found attached to *mba*24bp.1, as in UUR5; this TRU was also present in UUR2 and UUR8. Clinical isolate 4318 had 3 identifiable TRUs (*mba*24bp.1, *mba*276bp, and *mba*333bp). The conserved domain was attached to *mba*24bp.1. Isolate 4155 had 5 identifiable TRUs (*mba*24bp.1, *mba*45bp, *mba*213bp.2, *mba*252bp.1, and *mba*276bp). The conserved domain was attached to *mba*276bp; this TRU had not been previously seen attached to a conserved domain in any of the 14 ATCC type strains, including the clinical UPA3 described by Glass et al. [25]. This is a further confirmation that the TRUs found in the *mba* locus are part of this phase variable system, which trough recombination should be capable to present on the surface of the ureaplasma cell different TRUs at different times. It would be interesting to investigate whether some TRUs are more immunogenic than others and therefore may contribute to differential pathogenicity. As mentioned earlier the *mba* variable domain has been used as one of the determinants of serovar classification. It is interesting to note that serovars 4 and 12, which have an identical set of MBA genes, have a percent difference at the nucleotide level in a whole genome comparison (Table 3) of only 0.06 or 0.07% (value depends on which genome is used as reference sequence), making these serovars almost identical, with the exception of some minor rearrangements and small insertion/deletion events (see Additional file 2: Figure S5). In addition, we observed two chimeric *U. parvum* strains in a clinical isolate that had exchanged through horizontal gene transfer their *mba* genes [26]. Taken together, these observation suggest that the *mba* locus is dynamic and can comprise of a different set of variable domains at different times, therefore making this gene an unsuitable target for serovar differentiation.

## Conclusions

Ureaplasmas have been associated with many different clinical outcomes; however, they have been detected also in healthy individuals. Due to their differential pathogenicity, effort has gone into assignment of patient isolates into serovars and attempting to correlate specific serovars with specific clinical outcomes. Analysis of ureaplasma samples obtained from patients in the 1970s identified 14 different serovars based on patient and animal antiserum reactions. The expanded serotyping scheme developed by Robertson and Stemke in 1979 is based on antiserum generated by injecting rabbits with emulsified preparations of cell suspensions of each strain separately [59]. Studies were not done at this time to determine the antigen that the sera antibodies were recognizing. In a later study, Watson et al. (1990) reported the finding of an antigen recognized by infected humans that contains serovar-specific and cross-reactive epitopes. This antigen presented a multiple banded pattern on immunoblots, wherefore, it was named multiple banded antigen (MBA). The same study tested only 4 patient sera in blocking experiments with monoclonal antibodies; therefore, it is not possible to deduce the exact antigens for all serovars involved in the serotyping of the 14 serovars. Because of the suggested serovar-specific epitopes of the MBA, this protein has been used in attempts to develop better serotyping techniques. However, the cross-reactivity between serovars still could not be eliminated. Comparing the 14 genomes of the ATCC type serovars enabled us to better understand why there is cross-reactivity when attempting to use anti-MBA antibodies for serotyping. This is due to the fact that all ATCC serovars have more than two possible MBAs (when we include the genes in the locus that do not contain tandem repeats, as is the case of UUR13′s dominant *mba* gene), each expressed at different times, through a phase variable gene system. There was a limited number of unique variable domains, however, it was showed that one such unique variable domain unit was exchanged/acquired by horizontal gene transfer [26], suggesting that the *mba* locus is dynamic and can acquire or lose variable domains. Therefore the MBA genes are not suitable for a serotyping tool. Ureaplasmas have been shown to adhere to different eukaryotic cells although their adhesins have not been identified. Experiments done to gain a better understanding of the adhesion properties of ureaplasma showed that cytadherence involves N- acetylneuraminic acid (NANA) as a ligand receptor molecule. The same study showed that ureaplasma adherence was significantly lower, but not inhibited by neuraminidase treatment, therefore, there are additional unidentified receptors that do not involve NANA [60]. Our comparative genome analysis of the 14 ATCC serovars showed that ureaplasmas have a great variety of genes coding for surface proteins and lipoproteins.

Most of these genes could not be assigned a function, since they were orthologous to genes coding for proteins of unknown function or the predicted gene did not have an ortholog outside of the *Ureaplasma* genus. If these adherence related genes are of great importance to the organisms, our hypothesis suggests those genes will have a higher GC content than genes of lower importance. We used the %GC table together with signal peptide and transmembrane domain predictions to identify candidate genes that could be studied for adherence properties. A table of these genes can be found in the Additional file 3: Comparative paper COGs tables.xls, “Putative Surface Prot >27%GC” tab. The MBAs are part of the surface proteome of the ureaplasmas and have been shown to be recognized by the Toll-like receptors (TLR) and induce NF-κB production [52]. Recognition by the TLR can elicit the release of inflammatory chemokines and cytokines that in turn trigger prostaglandin production in the amnion, chorion, deciduas and myometrium, leading to uterine contractions and eventually may lead to pre-term labor. The variety of MBA variable domains and the capacity of the organism to vary their sizes and switch between variable domains could mean that different MBAs, when recognized by the TLRs, may have a different capacity to activate the innate immune system [61]. The fact that the MBA variable domain is recognized by patient antibodies and antibody pressure leads to phase variable switch in their size or the variable domain [53] suggests that the different variable domains could be used for host immune system evasion. Although we expected to find evidence of differential pathogenicity on the serovar level, the majority of the differences among the two species and the serovars are in genes encoding proteins for which we could not assign functions. There are a limited number of potential pathogenicity factors that could be recognized computationally. The previously shown activity of IgA protease in all 13 tested serovars [16,17,62] can be an important tool for host immune system evasion in the mucosal surfaces, however we could not identify the gene responsible for this enzyme activity computationally. The ureaplasmal IgA protease may be a novel IgA protease. We believe that one of the predicted genes, which contain a protease functional domain in their sequence may be responsible for the observed protease activity. PLC, PLA1 and PLA2 activity was also demonstrated previously [20,21,23] and has been thought to be a potential pathogenicity factor and contributor in adverse pregnancy outcomes. None of the genes encoding these enzymes was found in the 14 ureaplasma genomes computationally. Our attempts to detect PLC activity with a PLC commercial assay and by repeating the original experiments were unsuccessful.

Studies involving clinical isolates of ureaplasma have revealed hyper-variable DNA regions that may potentially harbor genes aiding the pathogenicity of ureaplasmas [34] and chimeric ureaplasma isolates revealing overwhelming evidence of extensive horizontal gene transfer in these organisms [26], which can explain the cross-reactivity of sera. Taken together these findings suggest that there might be innumerable serovars or strains based on different combinations of horizontally transferred genes. Our comparative genome study has identified genes that could support horizontal gene transfer. These genes combined with the observed chimeric clinical isolates of ureaplasma suggest that these organisms possess active recombination mechanisms. Therefore, it is possible that ureaplasmas do not exist as stable serovars in their host, but rather as a dynamic population. We do know that UUR causes non-gonococcal urethritis in males and pelvic inflammatory disease (PID) and/or endometritis in pregnant women more frequently than UPA; however no other clinical outcome is significantly more associated with either species or a particular serovar [26,63-68]. We cannot identify any clear gene or constellation of genes that might account for greater UUR virulence in some situations; although we do note a difference in the genes whose products are associated with resistance to H_2_O_2_, a known microbial pathogenicity factor. The widely different clinical outcomes of ureaplasmal infection could be the result of the presence or absence of potential pathogenicity factors in the colonizing ureaplasma strain. Alternatively, it may be more likely that the different clinical outcomes are either all or in part the result of patient to patient differences in terms of autoimmunity and microbiome.

Future studies of ureaplasma biology should concentrate on the development of molecular tools for the generation of ureaplasma gene knock-out mutants for example, in order to study genes potentially involved in pathogenicity. The sequenced genomes can aid in the development of such tools, by identifying transposons, integrated phage genomes, and genes involved in horizontal gene transfer. To aid the identification of potential pathogenicity factors, the large collection of clinical isolates should be explored for presence/absence of candidate genes. Considering the low cost of sequencing nowadays, the genomes of isolates from patients with different conditions should be sequenced and their comparison should further aid the identification of genes involved in differential pathogenicity.

## Methods

### Sequencing methods for ATCC and 4 clinical isolates

Ureaplasmas were grown in 10B medium and phenol chloroform extracted as described previously [25]. We randomly fragmented through shearing the purified genomic DNA from the 14 ATCC type strains and generated 1–2 kbp and 4–6 kbp fragment libraries. Using Sanger chemistry and ABI 3730 DNA sequencers, each serovar was sequenced to 8-12X redundancy. In order to obtain data to complete the genome sequence of Serovar 2, the Sanger data were supplemented with 454 pyrrosequencing (Roche) data. We sequenced the 4 clinical isolates only using 454 chemistry. Genome sequences produced with Sanger chemistry were assembled using the Celera Assembler. The 454 data were assembled using the Newbler Software Package for de novo genome assembly.

#### Annotation

All 14 ureaplasma strains were annotated using the JCVI Prokaryotic Annotation Pipeline followed by manual quality checks and manual curration to enhance the quality of annotation before being submitted to NCBI. Annotation was done on various levels, the individual protein level, the pathways and the multiple genome comparisons. The annotation pipeline has two distinct modules: one for structural annotation and the other for functional annotation.

The structural annotation module predicts an extensive range of genomic features in the genome. Glimmer3 [69] was used to predict the protein coding sequences whereas, tRNAs, rRNAs, cDNAs, tRNA and ribozymes are predicted based on matches to Ram libraries, a database of non-coding RNA families [70]. The programs tRNA scan [71] and ARAGORN [72], which is a program that detects tRNA and tmRNA genes. For functional annotation, JCVI uses a combination of evidence types which provides consistent and complete annotation with high confidence to all genomes. The automated annotation pipeline has a functional annotation module (AutoAnnotate), which assigns the function to a protein based on multiple evidences. It uses precedence-based rules that favor highly trusted annotation sources based on their rank. These sources (in rank order) are TIGRFAM HMMs [73] and Pfam HMMs, best protein BLAST match from the JCVI internal PANDA database and computationally derived assertions (TMHMM and lipoprotein motifs). Based on the evidences, the automatic pipeline assigns a functional name, a gene symbol, an EC number and Gene Ontology domains [74], which cover cellular component, molecular function and biological process(es). The assigned domains are related to evidence codes for each protein coding sequence with as much specificity as the underlying evidence supports. The pipeline also predicts the metabolic pathway using Genome properties [75], which are based on assertions/calculations made across genomes for the presence or absence of biochemical pathways. Genome properties incorporate both calculated and human-curated assertions of biological processes and properties of sequenced genomes. A collection of properties represents metabolic pathways and other biological systems and these are accurately detected computationally, generally by the presence/absence of TIGRFAMs and Pfam HMMs. This is the basis for the automatic assertions made for the presence of the whole pathway/system in any genome.

Finally a curator checked for consistency and quality of annotation, deleting spurious assertions and inserting any missed ones. This resulted in the manual merging of some genes, primarily the MBA genes, which were problematic for the automated genome annotation pipeline due to the nature of their repeats. JCVI’s internal Manual Annotation tool (MANATEE) [76] was used extensively to annotate these genomes. MANATEE is a freely available, open-source, web-based annotation and analysis tool for display and editing of genomic data. The genome comparisons and annotation transfer were done using the Multi Genome Annotation Tool (MGAT) which is an internally developed tool integrated within MANATEE to transfer annotations from one gene to other closely related genes. The clusters are generated based on reciprocal best BLASTP hits determined by Jaccard-clustering algorithm with a BLASTP identity > = 80%, a P value < = 1e-5 and a Jaccard coefficient threshold of 0.6. The clusters are composed of genes both within the genome and across different ureaplasma genomes. The same clusters are used in the genome comparisons generated by SYBIL ( http://sybil.sourceforge.net/), which is also an open source web based software package for comparative genomics [77].

#### Comparative genomics

The 19 genomes were compared using a variety of bioinformatics tools. Sybil [77] was used to generate clusters of orthologous genes (COGs), Jaccard clusters (paralogous gene clusters) and identify genes specific for each strain (singletons). The information generated with Sybil was used to deduce the pan genome for all 19 sequenced ureaplasma strains and different subsets of strains. PanSeq version 2.0 [78] was used to identify unique areas in the clinical UUR isolates that could not be serotyped. The functional annotation of genes in those areas was examined using MANATEE [76]. The percent difference table between pairs of genomes was generated by mapping pairs of ureaplasma genomes to each other using BLASTN; that is, contigs in genome 1 were searched against the sequences in genome 2. The BLASTN results were processed to compute the mean identity and fraction (of contig) covered for each contig in genome 1. These values were totaled to give the final value of mean identity and fraction covered when mapping genome 1 to genome 2. All 182 comparisons were carried out. In the mapping process, no attempt was made to compute a one-to-one mapping between genome 1 and genome 2, and thus, multiple regions in genome 1 can map to a region in genome 2. The mean percent difference was calculated from the generated data and reported in Table 3.

### MBA locus

The nucleotide sequence of all genomes was uploaded to the Tandem Repeats Database (TRDB) and the Inverted Repeats Database (IRDB) [79] and was analyzed using the tools in the database to find all tandem and inverted repeats. Genomes were analyzed one at a time and the main tandem repeating unit of the MBA of the serovar was located and the genomic area around it was inspected for other tandem repeats. This approach identified the presence of tandem repeats in the close vicinity to the MBA, that when compared through the Basic Local Alignment Search Tool (BLAST) [80] against the rest of the serovars’ genomes matched the MBA’s tandem repeating units of other serovars. The putative recombinase recognition sequence was identified by analyzing inverted repeats detected with the IRDB tools and close examination of the MBA loci of serovars 4, 12, and 13, which have the same set of tandem repeating units in different rearrangements. Dotplots were generated for these serovars using Dotter [81] and BLASTn [80] to help identify the conserved sequence that may serve as a recombinase recognition site. To identify other genes of the MBA phase variable system the all COGs generated by the Sybil [77] computes that had participating genes annotated as MBA were examined and organized into Figure 5.

#### PLC, PLA, and IgA protease genes

Tools used to search the genomes were BLAST [80,82] and Hidden Markov Models (HMMs) [83] deposited in PFAM [84]. We set up databases of all human ureaplasma open reading frames, proteins and full genome sequences. BLASTn and BLASTp [80,82] were used initially to search the open reading frames and protein databases with known PLC, PLA1, and PLA2 genes and protein sequences. Using this approach we were not able to identify any significant hits. To make sure that the gene was not missed by the gene predicting software, we used tBLASTn [82] to search the ureaplasma full genomes translated nucleotide database.

### PLC assay

Amplex® Red Phosphatidylcholine-Specific Phospholipase C Assay Kit (Invitrogen Cat.No.A12218) was used to detect activity of the enzyme in whole cell lysates, membrane, cytosolic, and media fractions of exponential and stationary phase cultures. The Amplex® Red Assay provides lecithin as substrate for PLC that when cleaved forms phosphocholine. Phosphocholine is modified to choline by alkaline phosphatase, which in the presence of choline oxidase produces betaine and H_2_O_2_. The Amplex red reagent in turn reacts in the presence of H_2_O_2_ and horseradish peroxidase to produce the red fluorescent compound resorufin. However, if the test sample contains PLD, PLD will cleave lecithin to produce choline, which bypasses the alkaline phosphatase step of the assay’s cascade; therefore, this assay would give a combined readout of PLC and PLD. Due to the potential presence of a PLD gene in ureaplasmas, to make the assay PLC specific we modified the assay by repeating it for each test sample, but omitting alkaline phosphatase from the reaction, in order to be able to subtract any activity by the putative PLD enzyme in the ureaplasma genomes. Everything else followed the manufacturer’s assay protocol. ATCC UPA3 and UUR8 cultures were grown in 10B or Trypticase Soy Broth to exponential phase. Cells were harvested through centrifugation and subjected to osmotic lysis. Cell membranes were collected through ultracentrifugation. The cleared cell lysates and the cell membranes were tested for PLC activity with the Amplex Red assay and with the previously published assay by DeSilva and Quinn [20,21,23].

#### Phylogenetic trees

Multiple sequence alignments (MSA) and phylogenetic tree constructions were performed using ClustalX 2.1 [85]. Phylogenetic trees were visualized with Dendroscope [86]. Multi-gene phylogenetic trees were generated by aligning the nucleotide sequences of 82 genes: the 7 genes encoding the urease subunits (*ureA-G)*, 47 genes encoding ribosomal proteins, 12 genes encoding RNA and DNA polymerase subunits, and 16 genes encoding tRNA ligases. The MSAs of all genes were concatenated and edited with Jalview 2.6.1 [87] to remove the non-informative positions (100% conserved in all 19 genomes) from the alignment. This was needed because the extreme similarity among the strains generated multiple sequence alignments containing approximately 5% informative positions. Although these informative positions were enough to separate the two species, they were not enough to resolve the relationship among serovars/strains within each species. The removal of the non-informative positions increased the bootstrap values but did not affect the structure of the clades. The phylogenetic tree was generated with ClustalX 2.1 neighbor-joining bootstrap option. The gene content tree was generated using the information from the formed clusters of orthologous genes (COG) to generate a table with a serovar on each row and a COG in each column. The presence of a gene in a serovar for each COG was marked with the number 0–6 (0 = none, 1–6 = number of copies of the gene in the serovar). Singletons were added to the table to increase the informative data. The core genome COGs (genes conserved in all 19 genomes) were removed from the dataset, since they are non-informative. To be able to use ClustalX 2.1 to generate the tree the numbers were turned to letters: (0 = C, 1 = S, 2 = T, 3 = P, 4 = A, G = 5, N = 6). The table was turned into a multifasta formatted file and loaded into ClustalX 2.1. The sequences did not need to be aligned with ClustalX 2.1, since they were already aligned. The tree was constructed using the bootstrap, neighbor joining method. The root for all trees is a poly-A sequence of similar size, since only the relationship within ureaplasmas was of interest.

## Authors’ contributions

VP performed the genome analyses, carried out the phospholipase assays, and was the primary author of this study. LBD, DMK, and LX prepared the ureaplasma samples, and consulted with the design of the sequencing study and analyses. JL, GHC and JIG did sequencing and analyses of the *mba* genes prior to the genome sequencing that influenced the analyses done on the genomes. SY, SS, JI, and JIG carried out some of the bioinformatics analyses and genome annotation. BAM coordinated the sequencing and conducted the assembly of the 14 ATCC type strains. GHC, KBW, and JIG conceived of the study, and participated in its design and coordination and helped to draft the manuscript. All authors read and approved the final manuscript. This project was funded with federal funds from the National Institute of Allergy and Infectious Diseases (NIAID), National Institutes of Health, Department of Health and Human Services under grants RO1A1072577 (VP, LBD, DMC, LX, JI, SY, KBW, JIG) and RR00959 (LBD, DMK, DMC, JL, GHC, JIG), and the NIAID Microbial Sequencing Program contract number N01-AI30071 (BAM, SS).

## Supplementary Material

Additional file 1 **Clinical isolates supplementary material.** Contains information about the relatedness of the four sequenced urealyticum clinical isolates to the ATCC stains and genes in their unique areas. Click here for file

Additional file 2 **Figures S1-S5.** Contains figures of additional phylogenetic trees. Click here for file

Additional file 3 **Comparative Genomics Tables.** Contains interactive tables of all gene clusters among the 19 ureaplasma genomes, % GC table, and a table of the genes from restriction modification systems in all 14 ATCC ureaplasma serovar strains. Click here for file

Additional file 4 **Table S1.** Contains anticodon table of tRNAs showing count of tRNAs used by human ureaplasmas. Click here for file

Additional file 5 **All Genes Encoding Recombinase or Transposase Proteins in All 19 Ureaplasma Genomes.** Contains a table of all genes in the 19 ureaplasma genomes that encode recombinase or transposase proteins. Click here for file
