# Supplementary material for: Comparative genome analysis of 19 Ureaplasma urealyticum and Ureaplasma parvum strains
Source: BMC Microbiol. 2012 May 30;12:88. doi: 10.1186/1471-2180-12-88 (PMC3511179; doi:10.1186/1471-2180-12-88)
Supplement: Additional file 1 — Clinical isolates supplementary material. Contains information about the relatedness of the four sequenced urealyticum clinical isolates to the ATCC stains and genes in their unique areas. [file 1471-2180-12-88-S1.doc]

**Genome Analysis of UU clinical isolates that could not be genotyped to the serovar level**

There were 67 (6%) isolates that were negative for all serovar-specific assays from the collection of 1061 clinical isolates of human ureaplasmas from diverse patient populations that we typed using our species and serovar specific real-time PCR assay. These serovars could not be assigned to any of the 14 known serovars by PCR [1]. Initial analysis of dotplots comparing each isolate’s genome to each ATCC serovar showed that isolates 2033 and 2608 were most closely related to serovars 12 and 4, which are the closest related serovars among the urealyticums (data not shown). Isolate 4155 was most similar to serovar 11, whereas isolate 4318 was most similar to serovar 2. All of the isolates’ genomes had some minor genome rearrangements, areas that were deleted, and some areas that were inserted and are new for the urealyticum group when compared to the ATCC reference strains. The reason these isolates were not positive for any of our serovar specific PCR assays was that the targets were either missing completely or some of the target was missing or modified so that one of the primers would not bind. However, it is clear that these isolates have changes in other areas of the genome as well. Whether we can assign new serovar numbers to any of the unidentifiable isolates is a matter of clarifying the requirements for a ureaplasma to be considered a specific serovar.

The genomes of the four isolates were examined for regions that were not present in any of the ATCC serovars using PanSeq [2]. Such areas were identified in isolates 2608 (3217nt long; genomic region coordinates: 4318-7534), 2033 (989nt long; genomic region coordinates: 585519-586507), and 4318 (3217nt long; genomic region coordinates: 21576-24792), but not in 4155. Isolates 2608 and 4318 had a 99% (3215/3217nt) identical region that was novel for ureaplasmas. All of these areas encoded hypothetical proteins of unknown function. The unique region of isolate 2033 unique area contained one 825 nt long gene (ORF00459). The gene encoded a signal peptide which suggests that it may be secreted. The unique area of isolate 2608 unique area contains 3 genes: ORF00004 (840nt), ORF00005 (561nt), and ORF00006 (1125nt). Neither of these genes has signal peptides or transmembrane domains. The unique area of isolate 4318 also contains 3 genes: ORF01501, ORF01502, and ORF01503.

References:

1. Xiao L, Paralanov V, Glass JI, Duffy LB, Robertson JA, Cassell GH, Chen Y, Waites KB: **Extensive horizontal gene transfer in ureaplasmas from humans questions the utility of serotyping for diagnostic purposes.** *J Clin Microbiol* 2011, **49:**2818-2826.

2. Laing C, Buchanan C, Taboada EN, Zhang Y, Kropinski A, Villegas A, Thomas JE, Gannon VP: **Pan-genome sequence analysis using Panseq: an online tool for the rapid analysis of core and accessory genomic regions.** *BMC Bioinformatics*, **11:**461.
