# Supplementary material for: Comparative genome analysis of 19 Ureaplasma urealyticum and Ureaplasma parvum strains
Source: BMC Microbiol. 2012 May 30;12:88. doi: 10.1186/1471-2180-12-88 (PMC3511179; doi:10.1186/1471-2180-12-88)
Supplement: Additional file 2 — Figures S1-S5. Contains figures of additional phylogenetic trees. [file 1471-2180-12-88-S2.doc]

**Supplementary Figure 1. Phylogenetic tree based on the gene content of the pan genome of ureaplasmas.**

**
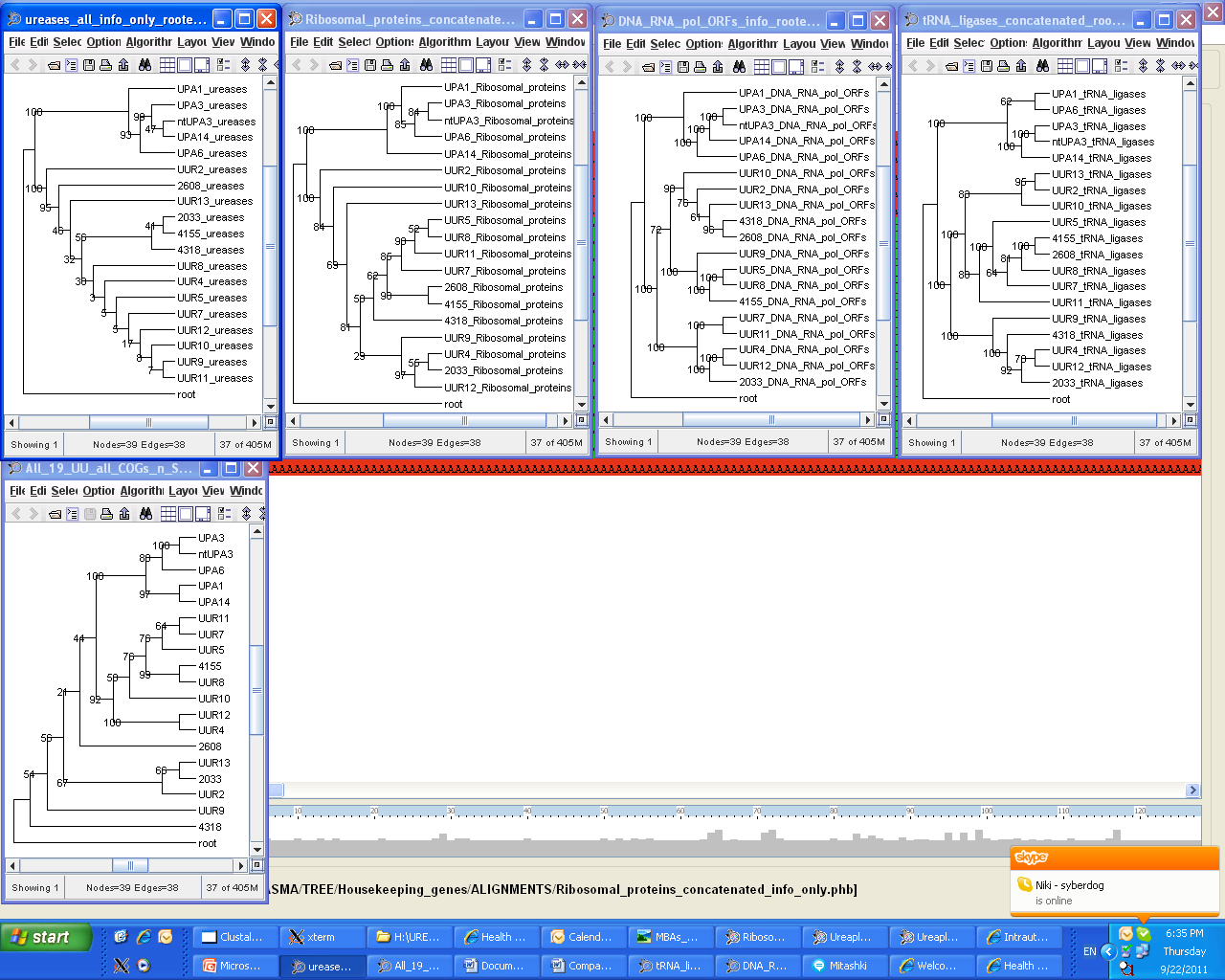
**

**Supplementary Figure 2. Phylogenetic trees based on the nucleotide sequence of multiple concatenated genes:** a. 7 urease genes; b. 47 ribosomal protein genes; c. 12 RNA and DNA polymerase genes; d. 16 tRNA ligases


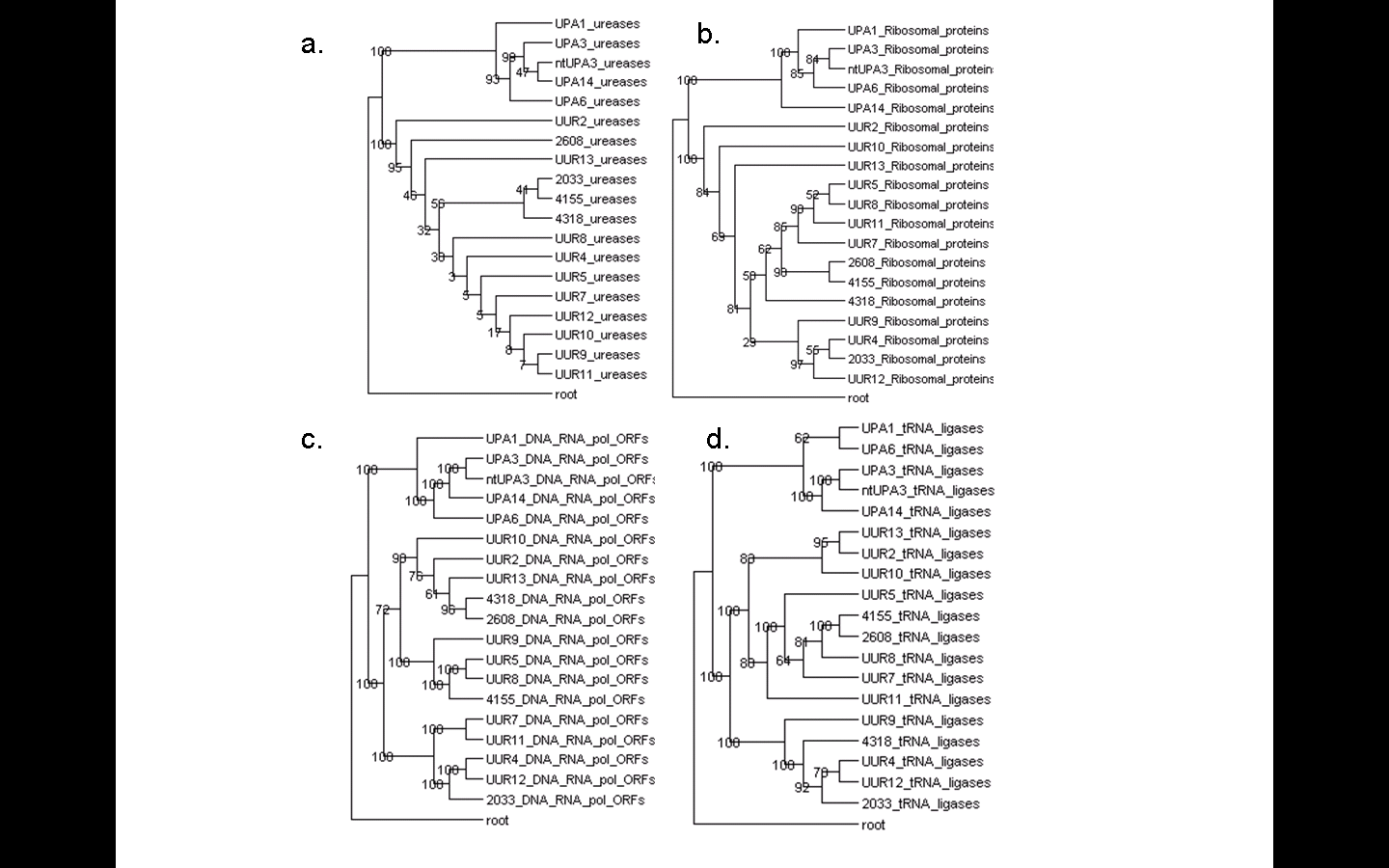


**Supplementary Figure 3. Phylogenetic trees based on the combination of two of the four groups of concatenated genes.** The nucleotide sequence of the genes was used; **a.** tree based on 7 usease genes and 12 RNA and DNA polymerase genes; **b.** tree based on 7 usease genes and 16 tRNA ligase genes; **c.** tree based on 7 urease genes and 47 ribosomal protein genes; **d.** tree based on 16tRNA ligase genes and 12 RNA and DNA polymerase genes; **e.** tree based on 16 tRNA ligase genes and 47 ribosomal protein genes; **f.** tree based on 12 RNA and DNA polymerase genes and 47 ribosomal protein genes.

**
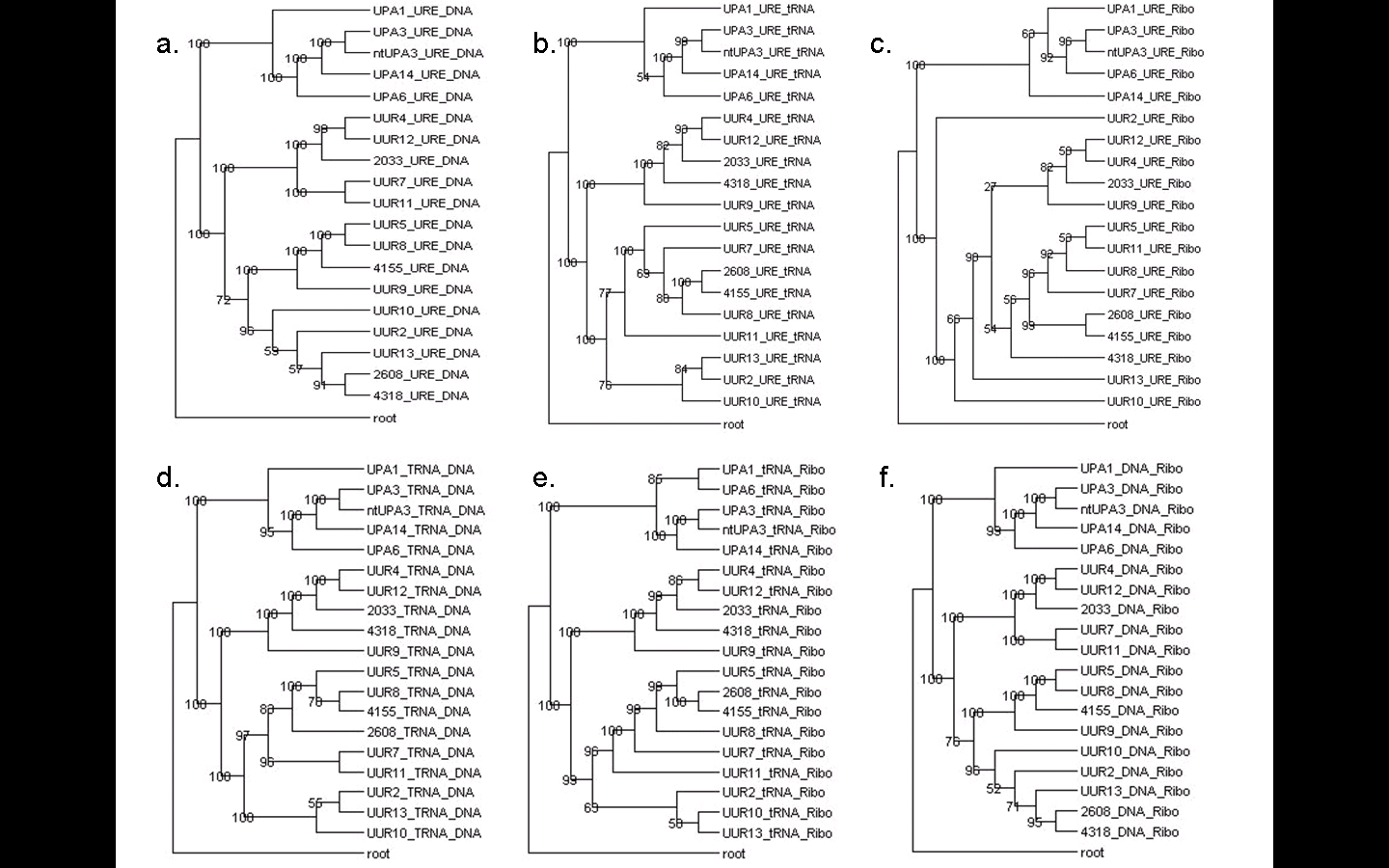
**

**Supplementary Figure 4. Phylogenetic trees based on the combination of tree of the four groups of concatenated genes.** The nucleotide sequence of the genes was used; **a.** tree based on 7 usease genes, 12 RNA and DNA polymerase genes and 16 tRNA ligase genes; **b.** tree based on 7 usease genes and 16 tRNA ligase genes, and 47 Ribosomal protein genes; **c.** tree based on 7 urease genes, 12 RNA and DNA polymerase genes, and 47 ribosomal protein genes; **d.** tree based on 16tRNA ligase genes, 12 RNA and DNA polymerase genes, and 47 ribosomal protein genes; **
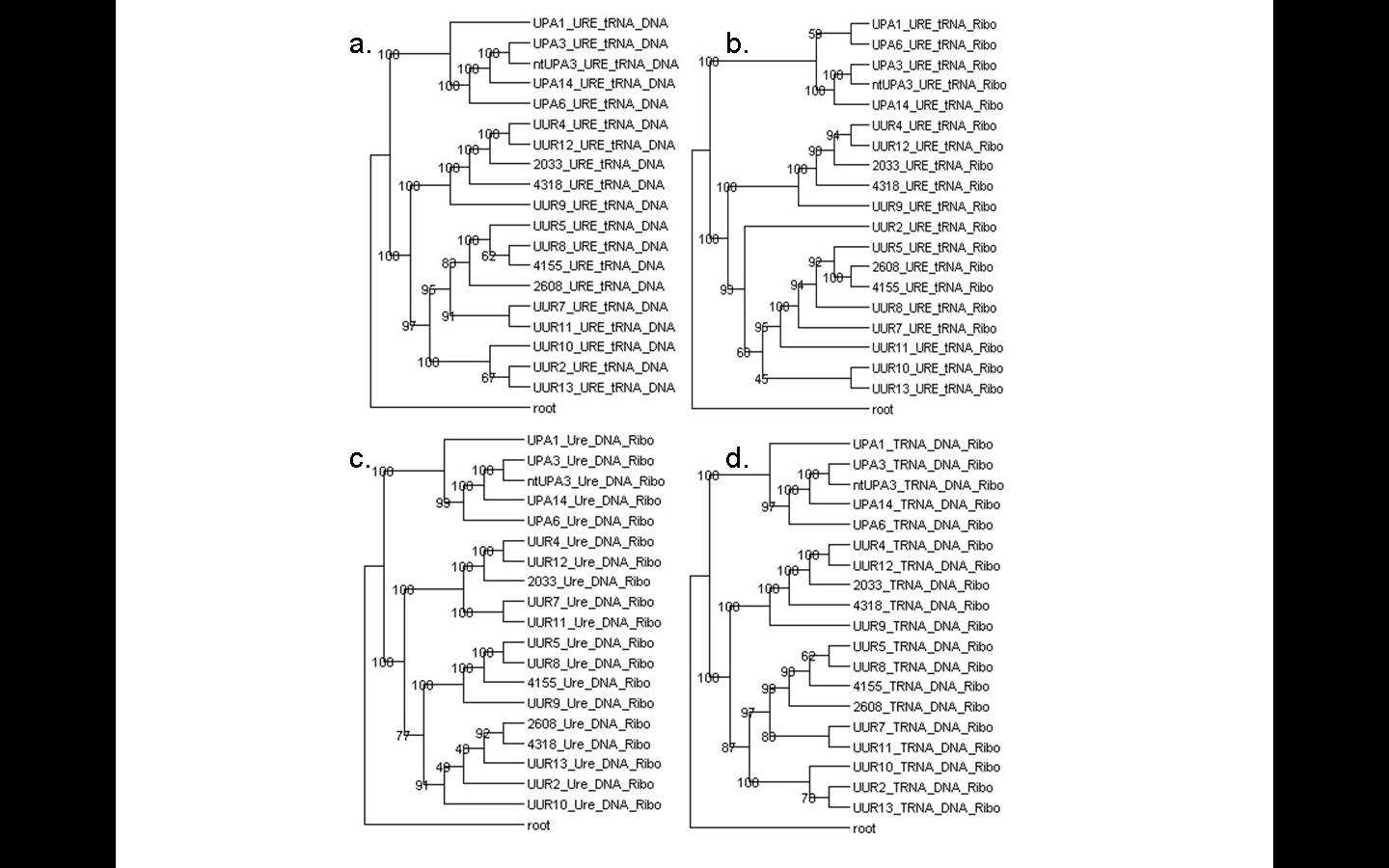
**

**Supplementary Figure 5. Dotplot of the genomes of UUR4 and UUR12.**

**
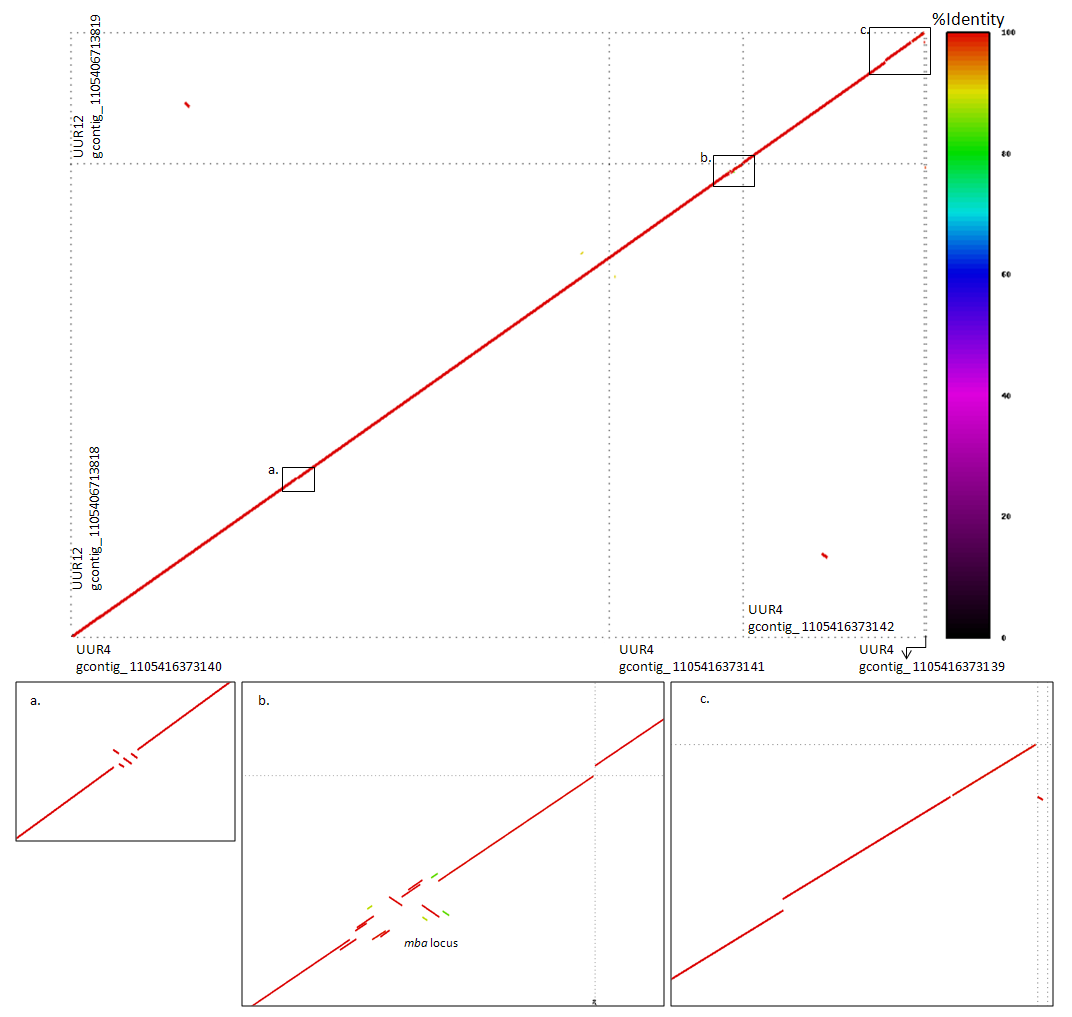
**
