# Supplementary material for: Comparative genome analysis of 19 Ureaplasma urealyticum and Ureaplasma parvum strains
Source: BMC Microbiol. 2012 May 30;12:88. doi: 10.1186/1471-2180-12-88 (PMC3511179; doi:10.1186/1471-2180-12-88)
Supplement: Additional file 4 — Table S1. Contains anticodon table of tRNAs showing count of tRNAs used by human ureaplasmas. [file 1471-2180-12-88-S4.doc]

**Supplementary Table 1. Anticodon Table of tRNAs Showing count of tRNAs used by Human Ureaplasmas.** The number next to each anticodon sequence represents the number of tRNA gene copies in ureaplasmas. Green marks the start anticodon and red marks the 2 stops. The UGA codon (a stop codon in the standard genetic code) codes for tryptophan and is read by tTrp_UCA_ in yellow on the table.

|  |  | Second Position | | | | | | | | | | | |  |  |
| --- | --- | --- | --- | --- | --- | --- | --- | --- | --- | --- | --- | --- | --- | --- | --- |
| First Position (5' end) |  | A | | | G | | | U | | | C | | |  |  |
|  | A | AAA | 0 | tPhe | AGA | 0 | tSer | AUA | 0 | tTyr | ACA | 0 | tCys | A | Third Position (3' end) |
|  | G | **GAA** | **1** |  | GGA | 0 |  | **GUA** | **1** |  | **GCA** | **1** |  |  |  |
|  | U | **UAA** | **1** | tLeu | **UGA** | **1** |  | UUA | - | Stop | **UCA** | **1** | tTrp |  |  |
|  | C | **CAA** | **1** |  | CGA | 0 |  | CUA | - |  | **CCA** | **1** |  |  |  |
|  | A | AAG | 0 | tLeu | AGG | 0 | tPro | AUG | 0 | tHis | **ACG** | **1** | tArg | G |  |
|  | G | GAG | 0 |  | GGG | 0 |  | **GUG** | **1** |  | GCG | 0 |  |  |  |
|  | U | **UAG** | **1** |  | **UGG** | **1** |  | **UUG** | **1** | tGln | **UCG** | **1** |  |  |  |
|  | C | CAG | 0 |  | CGG | 0 |  | CUG | 0 |  | CCG | 0 |  |  |  |
|  | A | AAU | 0 | tIle | AGU | 0 | tThr | AUU | 0 | tAsn | ACU | 0 | tSer | U |  |
|  | G | **GAU** | **1** |  | GGU | 0 |  | **GUU** | **1** |  | **GCU** | **1** |  |  |  |
|  | U | UAU | 0 |  | **UGU** | **1** |  | **UUU** | **1** | tLys | **UCU** | **1** | tArg |  |  |
|  | C | **CAU** | **3** | tMet | CGU | 0 |  | **CUU** | **1** |  | CCU | 0 |  |  |  |
|  | A | AAC | 0 | tVal | AGC | 0 | tAla | AUC | 0 | tAsp | ACC | 0 | tGly | C |  |
|  | G | GAC | 0 |  | GGC | 0 |  | **GUC** | **1** |  | **GCC** | **1** |  |  |  |
|  | U | **UAC** | **1** |  | **UGC** | **1** |  | **UUC** | **1** | tGlu | **UCC** | **1** |  |  |  |
|  | C | CAC | 0 |  | CGC | 0 |  | CUC | 0 |  | CCC | 0 |  |  |  |
